# Examining Relationships between Functional and Structural Brain Network Architecture, Age, and Attention Skills in Early Childhood

**DOI:** 10.1523/ENEURO.0430-24.2025

**Published:** 2025-07-24

**Authors:** Leanne Rokos, Signe L. Bray, Josh Neudorf, Alexandria D. Samson, Kelly Shen, Anthony R. McIntosh

**Affiliations:** ^1^Rotman Research Institute, Baycrest Health Sciences, Toronto, Ontario M6A 2E1, Canada; ^2^Department of Radiology, Cumming School of Medicine, University of Calgary, Calgary, Alberta T2N 2T9, Canada; ^3^Institute for Neuroscience and Neurotechnology, Simon Fraser University, Burnaby, British Columbia V5A 1S6, Canada; ^4^Department of Biomedical Physiology and Kinesiology, Faculty of Science, Simon Fraser University, Burnaby, British Columbia V5A 1S6, Canada

**Keywords:** functional connectivity, graph theory, neurodevelopment, neuroimaging

## Abstract

Early childhood is a critical period showing experience-dependent changes in brain structure and function. The complex link between the structural connectivity (SC) and functional connectivity (FC) of the brain is of particular interest. However, its relationship with both age and attention in early childhood is not well understood. In this study, children between the ages of 4 and 7, and at a 1 year follow-up visit, underwent neuroimaging (diffusion-weighted and passive-viewing functional magnetic resonance imaging) and assessments for selective, sustained, and executive attention. We examined regional graph metrics and SC–FC coupling of the structural and functional networks. Partial least squares was used to investigate longitudinal brain measure changes and cross-sectional associations with age and attention. We observed longitudinal changes in functional graph metrics and age-related decreases in SC modularity. Region-wise graph analyses revealed variable brain–behavior relationships across the brain, highlighting regions where structural topology is linked to age and attentional performance. Furthermore, we identified SC as a dominant predictor of age when compared with FC and SC–FC coupling. The findings emphasize how early childhood is a dynamic period where cognitive functioning is intricately and predominantly linked to structural network features.

## Significance Statement

This study investigates early childhood brain development, particularly the changes in structural and functional connectivity of the brain and the relationship between them. We examined children aged 4–7 over a year and used graph analyses to characterize variable developmental changes and brain–behavior relationships. Our findings emphasize the regionally specific relationship between brain network structure and behavior in early childhood and identifies critical regions (e.g., superior parietal lobule) where structural topology correlates with attentional performance. The identified regions can inform future work investigating potential targets for early intervention strategies in clinical populations. This research underscores the dynamic nature of childhood brain development and the predominant role of structural connectivity in cognitive maturation, providing insights into typical development.

## Introduction

Early childhood is a critical period of cognitive, behavioral, and brain development (Brown and Jernigan, 2012). Executive functioning skills, including working memory and attention, undergo intertwined development as children experience increasing and new attentional demands. Top–down attention skills have been classified into three attentional subsystems that demonstrate unique developmental changes (Zhan et al., 2010). These processes comprise sustained attention (i.e., “maintaining” attention for extended durations), selective attention (i.e., “orienting” attention for locating a target among distractors), and executive attention (i.e., “controlling” or shifting responses to changing circumstances; Manly et al., 2001; Breckenridge et al., 2013). Characterizing brain developmental changes underlying each attentional process has implications for understanding healthy and atypical development. A cross-sectional study with typically developing girls reported a link between distinct intrinsic functional connectivity (FC) networks and specific attentional abilities, as well as age-related FC network differences (Rohr et al., 2018). Across early childhood, there are also white matter (WM) changes, including alterations in mean, radial and axial diffusivity, fractional anisotropy, and more minor changes in streamline counts [i.e., structural connectivity (SC) measures; Lim et al., 2015; Dimond et al., 2020]. Diffusion-weighted magnetic resonance imaging (offering an indirect measure of WM microstructure) has been used to characterize nonlinear, regionally heterogeneous changes in WM tracts across the lifespan (Lebel et al., 2012, 2019; Reynolds et al., 2019). Longitudinal approaches for assessing functional and structural topology of early childhood brain networks will provide a more complete understanding of attentional processes in development.

Brain network topology assessed with graph analyses treats regions as nodes and structural and functional connections as edges (Bullmore and Sporns, 2009; Rubinov and Sporns, 2010). The brain appears to display a “small-world” topology, balancing segregation and integration for efficient information processing with high clustering characteristics (i.e., groups of nodes densely connected with their graph neighbors) and short paths (Watts and Strogatz, 1998). The brain's community structure (i.e., nodal organization into interconnected groups) can be assessed with a modularity measure quantifying how well a network can be separated into nonoverlapping modules (Newman, 2004). Clustering and modularity metrics have been used to characterize network topology in children, including high average clustering coefficients and modularity (Fair et al., 2009; Hagmann et al., 2010). Long-range axonal projection maturation is believed to underlie developmental increases in structural integration (e.g., more long-distance and intermodule connections) and decreasing segregation (e.g., fewer short-distance and intramodule connections; Hagmann et al., 2010; Tymofiyeva et al., 2013; Cao et al., 2017). Although functional networks reflect adult-like architecture by childhood, studies suggest ongoing refinement, with some functional networks becoming more segregated and others more integrated with age (Marek et al., 2015; Grayson and Fair, 2017; Pines et al., 2022). Variable patterns of segregated and integrated functional networks may depend on the network's position on the sensorimotor-association axis (Keller et al., 2023), highlighting the importance of considering region-wise metrics when investigating the relationship between network development and attentional skills.

The relationship between SC and FC is also of immense interest (see Suárez et al., 2020 for review). Studies suggest the relationship between SC and FC strengthens across the lifespan (Hagmann et al., 2010; Betzel et al., 2014). SC–FC coupling has also been found to be a stronger predictor of age (between 18 and 82 years) compared with SC or FC alone (Zimmermann et al., 2016). Conversely, there are reports that SC had limited impact on the decline in FC with aging (ages 17–79; Madden et al., 2020). Recent work has further suggested heterogeneous structure–function relationships across the brain (ages 7–85; Zamani Esfahlani et al., 2022). Therefore, studying both SC and FC changes can help elucidate healthy neurodevelopmental patterns and potential predictive factors of behavioral outcomes.

In this study, we investigated structural and functional networks of children between the ages of 4 and 7 and at a 1 year follow-up visit. We used partial least squares (PLS) to assess if regional graph metrics and SC–FC coupling changed over the follow-up (longitudinal) and to explore if the brain measures were related to age and attention (cross-sectional behavior/age associations at two time points). We focused on network segregation metrics to aid interpretability for FC data. Integration metrics (e.g., global efficiency) that depend on shortest path lengths are difficult to interpret given the contribution of indirect connectivity in FC (Rubinov and Sporns, 2010). In a combined analysis, we compared SC, FC, and SC–FC coupling with age. We hypothesized that older age and better attention skills would be associated with lower, region-specific segregation and greater SC–FC coupling (Hagmann et al., 2010), supporting more efficient and coordinated communication between brain regions.

## Materials and Methods

### Participants

Data were collected from typically developing children (4–8 years) at Alberta Children's Hospital. All participants provided written informed consent (parents) and assent (children). The study received approval by the Conjoint Health Research Ethics Board of the University of Calgary. Exclusion criteria for potential participants included history of neurodevelopmental or psychiatric disorders; neurological diagnosis; and chronic medical condition or gestational age <37 weeks. The inclusion criteria were participants who had three neuroimaging scans (i.e., T1 weighted, multishell dMRI, and fMRI scans). Following neuroimaging processing and quality control (QC) exclusion criteria described below, the final sample included 39 participants (20 girls) with an initial scan (mean age, 5.76 [4.14–6.88]) and a scan at 1 year follow-up (mean age, 6.85 [5.13–7.89]; i.e., 78 scans). Four participants were reported to be nonright-handed by their parents including left-handed (*n* = 1), more left-handed at time point one and left-handed at follow-up (*n* = 1), and ambidextrous (*n* = 2) at time point one but more right-handed at follow-up.

### Data collection

During two distinct sessions (2 h) within a 2 week period, both cognitive assessments and MRI scans were conducted. The initial session consisted of a number of attention tasks that were administered in a randomized order (within and between sessions). The participants also underwent training in an MRI simulator whereby they practiced lying down in the scanner while listening to MRI scanner sounds through headphones. During the practice scan, they also viewed the same 18 min video shown in the real MRI scanner, such that children had similar familiarity with the video at the baseline and follow-up scans. The actual MRI scanning process and the remaining attention tasks were conducted during the second session. The attention tasks were completed in a testing room adjacent to the MR simulator.

### Assessment of attention skills

Participants’ attention skills were assessed with four tasks including measures of sustained attention (visual and auditory), selective attention, and executive attention. The assessments were modeled on the Early Childhood Attention Battery (Breckenridge et al., 2013), which was developed as an adaptation of the Test of Everyday Attention for Children (Manly et al., 2001) suitable for children aged 3–6 years old. All assessments were administered using a Dell laptop computer (screen size, 31 × 17.5 cm; viewing distance, 35–50 cm), except for the selective attention subtest (see below, Selective attention task). External speakers were used to play auditory items. To ensure the child comprehended the instructions for each computer-based task, a practice trial was completed and repeated if necessary.

#### Sustained attention tasks

The visual sustained attention task included the presentation of a continuous sequence of pictures, with each image appearing for 200 ms [interstimulus interval (ISI), 1,800 ms]. Thirty target images (an animal) and 120 nontargets (common everyday items) were presented. When a target appeared, the child's task was to respond “yes,” “animal,” or with the name of the animal. If four consecutive targets were missed, the child received a prompt to maintain their attention. The measure was scored as the sum of correct responses, minus errors and prompts.

The auditory sustained attention task included a continuous sequence of words (average duration, 650 ms; ISI, 1,350 ms). Both monosyllabic target (animal) and nontarget (familiar item) words were presented. The child's task was to respond “yes,” “animal,” or with the name of the animal when they heard a target word. The number of correctly identified targets, minus errors and prompts, was taken as the auditory score. Lastly, the total sustained attention score was calculated as the mean of visual and auditory sustained attention scores.

#### Selective attention task

In the selective attention task, children were presented with a laminated letter-sized search sheet containing both targets (18 red apples) and distractors (162 white apples and red strawberries). They were given 60 s to point to the targets and an experimenter marked correctly identified targets with an erasable marker. The score was the sum of correctly identified targets.

#### Executive attention task

A child-appropriate Wisconsin Card Sorting Test adaptation was used for the executive attention task (Robinson et al., 1980). The child's task was to determine which type of balloon a teddy bear preferred. The bear liked a specific color of balloon in Stage 1 and a different color in Stage 2. In Stage 3, the bear liked a specific shape of balloon. Visual feedback (and no other information) was given to the children on whether they made the correct choice. Six consecutive correct responses (out of 20 possible trials for each stage) were required to pass. The test was discontinued if a child failed a stage. The executive attention task score was the total number of incorrect or incomplete trials (multiplied by negative 1 so that more positive values reflected better attention for all analyses).

#### Behavioral analyses

The statistical analyses were conducted using MATLAB Version 9.11 (R2021b) and R version 4.3.0 (R Core Team, 2023). The relationships between age (fixed effect) and attention skills were computed with the “lme4” package in R (Bates et al., 2015) to account for repeated measures (i.e., participants’ intercepts specified as random effects). The Satterthwaite method (“lmerModLmerTest”) was used to calculate the *p* values for these models (Hrong-Tai Fai and Cornelius, 2007; Kuznetsova et al., 2017). Wilcoxon signed rank tests were conducted to explore whether there were differences in the performance of each attention assessment between time points. For only a single participant that had a sustained attention score >3 standard deviations from the mean, a single attention assessment (auditory) was used to calculate the average score, consistent with prior methodology (Rohr et al., 2018). Additionally, one participant did not complete the executive attention task, and thus the missing value was replaced with the sample's average score.

### Neuroimaging data and preprocessing

Participants underwent an MRI scan while awake and watching clips from a children's television show (Elmo's World). The scans were collected on a 3 T General Electric MR750w scanner (GE Healthcare), with a 32-channel head coil. FMRI data were acquired with a gradient echoplanar imaging (EPI) pulse sequence [TR/TE, 2,500/30 ms; FA (flip angle), 70; FOV, 64 × 64 mm; number of slices, 34; resolution, 3.5 mm isotropic voxels; number of volumes, 437]. T1-weighted imaging data were acquired with a FSPGR BRAVO sequence (TR/TE, 6.764/2.908 ms; FA, 10; FOV, 512 × 512 mm; number of slices, 226; resolution, 0.8 × 0.8 × 0.8 mm isotropic voxels; scan time, 577 s). Diffusion-weighted images were acquired using a 2D spin-echo EPI sequence (TR/TE, 1,000/86 ms; FA, 90; FOV, 230 × 230 mm; resolution, 2.5 mm isotropic voxels; slices, 45; 3 interspersed *b* = 0 s/mm^2^ volumes; *b* = 1,000, 2,000 s/mm^2^; number of directions, 45; distributed on a whole-sphere).

The MRI data were preprocessed with TheVirtualBrain-UK Biobank pipeline (Frazier-Logue et al., 2022), which was adapted for pediatric populations. This multimodal (anatomical, fMRI, dMRI) processing pipeline primarily relies on the FMRIB (Functional MRI of the Brain) Software Library toolbox (Jenkinson et al., 2012) to generate the SC and FC matrices used in subsequent analyses. The pipeline also produces detailed QC reports of raw, intermediate, and processed outputs of the pipeline, supporting the extensive manual QC of pediatric data. The pipeline steps are described below.

#### Structural processing subpipeline

Preprocessing included an initial brain extraction with optiBET, an optimized brain extraction tool that results in high-quality, robust brain extraction for brains with severe pathology (Lutkenhoff et al., 2014). Processing also included a nonlinear registration of the T1-weighted images to an age-specific template (Fonov et al., 2011), NIHPD asymmetrical (natural pediatric template optimized for ages 4.5–8.5 years), followed by bias correction and segmentation. A 200 cortical region-of-interest (ROI) parcellation (Schaefer et al., 2018) was transformed into the NIHPD template space and was registered to the T1-weighted images. Segmentation of WM, gray matter (GM), and cerebrospinal fluid signals was completed using FSL's FAST. The WM and GM segmentations were used to create WM interface masks (i.e., the WM voxels bounding the GM) and exclusion masks.

#### Functional MRI processing subpipeline

For the fMRI data, brain extraction, motion correction (MCFLIRT), slice timing correction, spatial smoothing (FWHM,4 mm), high-pass filtering (100 s), and registration (to the T1-weighted image and age-specific template) were completed using FSL's FEAT toolbox. Manual classification of noise and signal components from FSL's MELODIC ICA was completed and validated by two other lab members (K.S., A.D.S.) to create a training set of 23 participants that were representative of the dataset (e.g., matched for sex, time point; Griffanti et al., 2017). The training set included 13 female and 10 male participants (M_age_ = 6 years [4.26–7.89]; SD = 1.00). FSL's FMRIB's ICA-based Xnoiseifier (FIX) with “aggressive” artifact removal (Griffanti et al., 2014) was used to perform denoising (i.e., all noise components and motion confounds were regressed). The aggressive approach removes the total shared variance between signal and noise and was adopted due to the presence of high levels of motion. The ROI parcellation registered to the T1-weighted images (i.e., as described above and output from the structural subpipeline) was registered to a reference fMRI volume.

#### Diffusion MRI processing subpipeline

The diffusion data processing entailed B0 field estimation and unwarping using the Synb0-DisCo tool (Schilling et al., 2019) to help improve T1-weighted and dMRI image registrations. Specifically, the Synb0-DisCo tool created a synthetic undistorted B0 image for dMRI distortion correction (i.e., input into FSL's TOPUP toolbox). Head motion and eddy correction (EDDY), brain extraction (BET), and registration of the templates, interface, and exclusion masks to DTI space were also completed. Diffusion tensor fitting (DTIFIT) with the FMRIB's Diffusion Toolbox (FDT) was used to calculate the diffusion tensor model of each voxel to check the fiber directions.

FDT's BEDPOSTX was also implemented to model the crossing of fibers in each voxel and distributions of diffusion parameters by using Bayesian estimation (i.e., Markov chain Monte Carlo sampling). Probabilistic tractography using PROBTRACKX2 (fiber volume threshold, 0.01; number of steps, 2,000; step length, 0.5 mm; number of samples, 5,000; loop check, on) was then completed. Specifically, PROBTRACKX2 takes many samples from the voxel-wise diffusion parameter distributions to create a histogram of the number of streamlines connecting each ROI pair. The interface masks (created previously as described above, Structural processing subpipeline) were used to prevent overdefining WM structures. The ROI masks were used as seeds, while the GM exclusion masks were used to exclude any streamline that entered the masked region. For a particular ROI, their streamlines terminated when they reached any of the other ROI masks.

#### Connectivity matrices

The FC matrices were created for each participant by calculating the Pearson's correlation coefficient of the BOLD time series for each pair of regions. The FC matrices were then Fisher *Z*-transformed. The SC weights matrix for each participant consisted of the number of streamlines between ROI pairs divided by the total number of streamlines sent from the seed ROI. The matrices were symmetrized (i.e., the weights were averaged over both directions) as the tractography approach does not provide information about connection directionality. Extended Data Figure 2-1 documents the ROI name abbreviations for the ROIs assessed for all region-wise analyses. The raw dMRI images’ FOV were consistently missing seven ROIs (Extended Data Fig. 2-2) due to regions being cut off at the bottom of the image stack; therefore those regions were excluded and the resulting 193 × 193 ROI functional and SC matrices were used for network analyses. No ROIs were excluded due to fMRI data quality. Consensus thresholding was applied to the SC matrices to address false positives that can result from probabilistic tractography (Shen et al., 2019). Specifically, connections were set to zero if they were not present in at least 75% of the high-quality dMRI scans (i.e., scans that passed the QC steps described below; *n* = 176; De Reus and Van Den Heuvel, 2013). On average, 6,745 connections (18.1%; SD = 1,671.3) were set to zero.

#### QC

**Figure 1.**
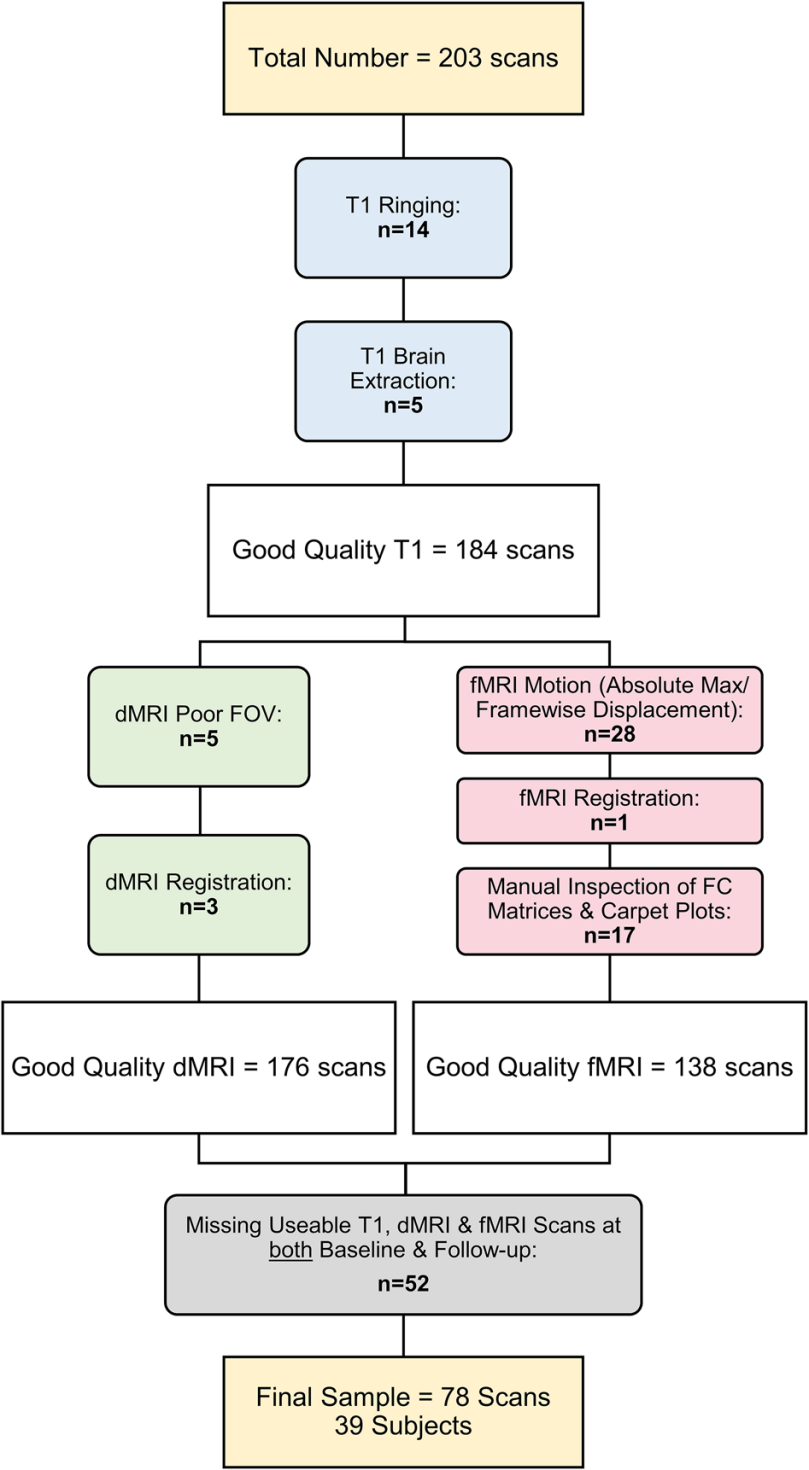
Exclusion and inclusion criteria. The initial dataset included 203 scans. The respective numbers of excluded scans are documented in bold. After QC of the T1 images (blue), there remained 184 good-quality T1 images. The dMRI (green) and fMRI (red) scans were then assessed, resulting in 176 and 138 good-quality scans, respectively. Lastly, subjects without good-quality T1, dMRI, and fMRI scans at both baseline and follow-up (i.e., three usable scans at each time point) were excluded from the final analyses. The resulting final sample included 39 subjects with scans at two time points, totaling 78 scans. FOV, field of view; FC, functional connectivity.

QC of the neuroimaging data (*n* = 203 scans) was completed, including manual inspection of each participant's brain extraction, segmentation, registrations, masks, fiber directions, tracts, SC matrices, cleaned fMRI ROI time series, and FC matrices (Fig. 1).

Participants’ T1 images with the presence of extreme motion artifacts (i.e., ringing) and poor brain extraction were noted and excluded (*n* = 14 and *n* = 5, respectively) with consideration of downstream functional and diffusion subpipeline outputs (e.g., the impact on registration). Additionally, scans with extremely poor FOV (e.g., missing additional regions to those excluded at the group level; see above) were excluded (*n* = 5).

Scans with poor dMRI-to-T1 registration were also excluded (*n* = 3). The SC matrices, along with the distribution of weights and tract lengths, were inspected for extreme sparsity. No other participants were excluded based on their SC matrices.

FMRI scans with >8 mm maximum absolute displacement or >0.3 mm framewise displacement for >8 min were excluded (*n* = 28). Participants with extremely poor fMRI-to-T1 registration were noted and in conjunction with inspection of their FC matrices were excluded (*n* = 1). More specifically, the FC matrices were checked for weak homotopic connectivity, indistinguishable intra- and interhemispheric quadrants, and significant “banding” (i.e., an indication of motion artifacts or poor registration). The impact of residual motion artifacts was assessed with the MCFLIRT displacement plots and carpet plots of the ROI time series after cleaning. Scans with extreme residual motion based on the manual inspection of the FC matrices and carpet plots were excluded (*n* = 17).

Lastly, participants who did not pass QC on all three neuroimaging data types (T1, fMRI, dMRI) for both a baseline and 1 year follow-up visit were excluded (*n* = 52), ensuring an equal number of subjects in each condition (i.e., a requirement for subsequent analyses described below, PLS analyses). Therefore, a total of 125 scans were excluded after all QC checks, resulting in a final analyzed sample of 78 scans from 39 participants.

A number of additional QC assessments were completed on the final sample (*n* = 78). Reports from the eddy current correction step (EDDY) indicated that the final analyzed scans had no >3.5% total outliers (e.g., from motion causing signal dropout). After fMRI FIX cleaning, the temporal signal-to-noise ratio (tSNR) was not significantly different between time points (*t*_(38)_ = −1.39; *p* = 0.17). A linear mixed-effect model with participants’ post-FIX tSNR values and random intercepts for each participant (i.e., to account for within-participant variations) also found no significant relationship with either handedness, age, mean relative head motion, or mean absolute head motion (*p*'s > 0.26). For the final sample, there were also no significant differences in the mean absolute head motion between time points for the dMRI (*t*_(38)_ = 2.02; *p* = 0.77) or fMRI data (*t*_(38)_ = 2.02; *p* = 0.20). In addition, there were no significant differences in the mean relative head motion between time points for the dMRI, (*t*_(38)_ = −1.20; *p* = 0.24) or fMRI data (*t*_(38)_ = 0.65; *p* = 0.52). A QC-FC metric, the percentage of FC edges that correlated with head motion (average framewise displacement; *p* < 0.05 uncorrected; Ciric et al., 2017; Graff et al., 2022a; Parkes et al., 2018), was calculated to be only 16.8% (median *R* = 0.075) for the final sample.

#### Fingerprinting

As an indication of whether the adopted preprocessing cleaning approach of the functional data decreased or enhanced individual-specific information (i.e., to assess the impact of the preprocessing approach), we used a fingerprinting match rate approach described previously (Graff et al., 2022a). This metric was computed as the number of times an fMRI scan had the highest correlation with a second scan from the same participant, divided by the total number of scans (*N* = 138; effectively comparing the correlations within subject to between subjects). We calculated the fingerprinting match rate on all 138 high-quality fMRI scans, including scans that did not have two high-quality time points. These scans served as potential false matches. The match rates as percentages for the scans after preprocessing compared with no confound mitigation were 82.22 and 11.11%, respectively. Previously, Graff et al. (2022a) achieved a maximal match rate of 90.2% on an overlapping sample.

### Network analysis

The Brain Connectivity Toolbox (https://sites.google.com/site/bctnet) was used to calculate the metrics for network analysis. Modularity, local clustering, and average weighted degree were chosen to characterize the network topology (e.g., segregation) in the developing brain.

The matrices’ diagonals (i.e., self-connections) were set to zero, and negative weights were retained in the FC matrices. Modularity (*Q*) was calculated using the “community_louvain.m” function. Asymmetric treatment of negative weights was used for the FC matrices, such that a greater contribution was given by the positive weights (Rubinov and Sporns, 2011). Subjects’ *Q* values were also compared with 1,000 null models that were generated with the “null_model_und_sign” function (i.e., preserved weight and degree distribution, approximated strength distributions) for each participant. For all participants, each individual's SC and FC networks were significantly more modular than the null models (*p*'s < 0.001), indicating that the modules identified are statistically different from chance and therefore meaningful to analyze. In order to assess changes in modularity with age, a linear mixed-effect model with participants’ SC modularity statistic was tested with age, handedness, sex, visit (as a factor), dMRI absolute and relative motion as fixed effects, and random intercepts for each participant. Similarly, a model for participants’ FC modularity statistics with age, handedness, sex, visit, and fMRI absolute and relative mean displacement was also tested. Linear models for each attention measure were also run with the differences in *Q* between the two visits as the dependent variable. These models included the difference in age, attention, and motion, as well as baseline sex and handedness as fixed effects.

The local clustering coefficient is the ratio of the connections between a region's neighbors (i.e., observed triangles) and the total possible number of such connections (Watts and Strogatz, 1998) and was calculated with the “clustering_coef_wu.m” (SC) and “clustering_coef_wu_sign.m” (FC; Constantini and Perugini's generalization) functions. Subjects’ local clustering coefficient values were also compared with 1,000 null models (generated with the “null_model_und_sign” function) for each participant. The local clustering was significantly greater than the null models (*p*'s < 0.05) for 98.6% of the 15,054 (193 regions × 78 subjects) SC network regions and in 96.8% of the FC network regions.

For each participant's SC matrix, average “weighted degree” was also calculated by taking the column-wise “average connection strength” for each region, resulting in a 1 × 193 SC vector (where 193 is the number of regions; Rubinov and Sporns, 2010; Zimmermann et al., 2016). This was also completed for the participants’ FC matrices.

### SC–FC coupling

A SC–FC coupling metric was calculated as the Spearman's correlation between a region's SC and FC profiles for each participant at each time point, following the approach described by Zimmerman et al. (2016). This resulted in a single SC–FC coupling value for each ROI.

### PLS analyses

**Figure 2.**
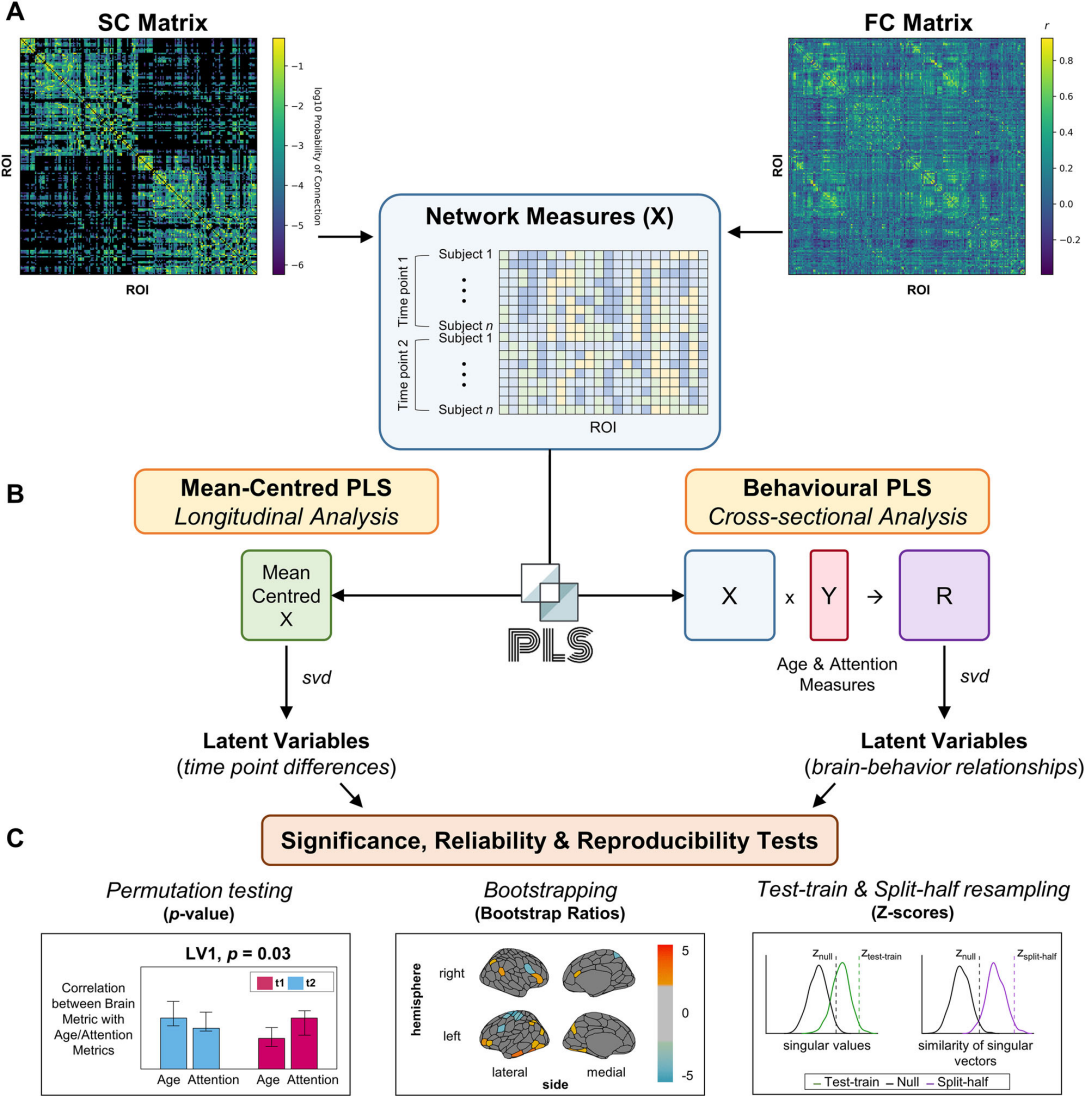
Schematic of analyses. ***A***, The SC (log-transformed for visualization) and FC matrices (shown for a representative subject) were used to compute brain network measures (X) including modularity, local clustering, weighted degree, and SC–FC coupling. For each measure, the resulting vectors were stacked into a subject × ROI matrix, with Condition 1 (Time Point 1) stacked above Condition 2 (Time Point 2). The ROI names of the parcellation and the ROIs excluded from all analyses are outlined in Extended Data Figures 2-1 and 2-2, respectively. ***B***, For each brain measure, mean-centered PLS analyses were conducted to identify LVs that characterize time point difference (longitudinal analyses). The mean-centered PLS analysis computes a SVD on the mean-centered brain data (left). bPLS analyses were also performed to identify LVs characterizing brain–behavior associations with age and each attention measure (cross-sectional analyses at two time points). The bPLS analysis computes the SVD on the correlation matrix between the brain and behavioral (Y) data (right). ***C***, Significance, reliability, and reproducibility tests were then conducted with permutation testing, bootstrapping, and test-train and split-half resampling, respectively. The associated *p* values for each LV (left) were identified using 1,000 permutations. Bootstrapping with 500 iterations was used to identify brain regions that reliably exhibited the identified relationships and the reliable BSRs (thresholded at +2.0 and −2.0) were plotted on a brain (middle). Lastly, two reproducibility assessments (right) were conducted with split-half resampling (500 resamples) to assess the reproducibility of the singular values and singular vectors across various sample sizes (“test-train” and “split-half,” respectively). The distributions of the singular values (green) and similarity of singular vectors (purple) were used to calculate *z*-scores. The *z*-scores were then compared with those from null distributions generated through permutation resampling, where a difference greater than ±2 suggests that the result is reproducible.

PLS correlation analyses (McIntosh and Lobaugh, 2004; Krishnan et al., 2011) were conducted to identify maximal covariance patterns in the data, known as latent variables (LVs). PLS is particularly advantageous as it is able to cope with collinearity among the variables. Specifically, two-condition mean–centered task PLS analyses and behavioral PLS (bPLS) analyses were conducted. A schematic of the analysis workflow is detailed in Figure 2. For a tutorial and detailed examples, we refer readers to Krishnan et al. (2011) and McIntosh and Lobaugh (2004), which provide comprehensive explanations and applications of PLS for neuroimaging.

10.1523/ENEURO.0430-24.2025.f2-1 Figure 2-1 Parcellation region abbreviations and full names. Download Figure 2-1, DOC file.

10.1523/ENEURO.0430-24.2025.f2-2 Figure 2-2 Region name and parcellation number for the regions excluded from the analyses. Download Figure 2-2, DOC file.

Mean-centered task PLS uses a mean-centered matrix calculated from the brain observations (specified as *X*) with two conditions (i.e., visits). This “task” PLS identifies within-subject brain changes across the 1 year follow-up, i.e., a longitudinal analysis.

bPLS analysis first computes a correlation matrix between a particular brain measure (specified as *X*) and the behavioral measures (specified as *Y*). The bPLS identifies whether the brain–behavior correlations are similar at time points one and two, i.e., a cross-sectional analysis that includes two time points assessed in parallel.

The calculated data matrix (*X*) is decomposed using singular value decomposition (SVD) as follows:

$$X=USV^{T}.$$

Mutually orthogonal LVs are defined by a left singular vector (*U*), a right singular vector (*V*), and a singular value (*S*). The singular vectors, also known as saliences, represent the contribution of the *X* and *Y* variables for each LV. Specifically, *U* contains the brain saliences and *V* contains the task/behavior saliences. The singular values help communicate the covariance between *X* and *Y* that is explained by a given LV.

#### Matrices for PLS analyses

The brain measures analyzed were the modularity statistic, local clustering coefficients, regional average connection weights (i.e., weighted degree), and SC–FC coupling values. For a given region-wise brain measure, the vectors for each participant at time point one were stacked resulting in a matrix with dimensions 39 participants × 193 regions. This was also done for the vectors for participants at time point two. The resulting matrix for time point two was appended to the time point one matrix, resulting in a 78 scan (i.e., 39 participants × 2 scans) by 193 region matrix (Fig. 2*A*). The same steps were completed for each network metric and the SC–FC coupling values. Mean-centered task PLS analyses were conducted for each brain measure between the two visits (Fig. 2*B*, left). bPLS analyses were performed for each brain measure with age and attention measures (Fig. 2*B*, right).

An additional bPLS analysis was conducted with the SC and FC weighted degree, as well as the SC–FC coupling (at the baseline and follow-up). The SC, FC, and SC–FC matrices were all of dimensions 78 scans × 193 regions and were stacked to create the resulting brain data matrix (234 scans × 193 regions).

#### Tests of specificity

The cosine similarity of the brain saliences from each respective task PLS analysis and bPLS were calculated to identify whether the regions that were different between time points (i.e., identified by the task PLS) were the same as those that were correlated with age and behavior (i.e., identified by the bPLS). This was meant as a qualitative assessment only.

Furthermore, separate two-condition bPLS analyses were conducted with each brain metric with sex and motion metrics (i.e., mean relative fMRI motion and percentage of dMRI outliers for FC and SC metrics, respectively). Both motion metrics were used for the SC–FC coupling metric analyses. The cosine similarity of the brain saliences from each PLS analysis and the respective bPLS analyses with sex and motion were calculated. This was conducted to assess whether the regions identified by the task PLS and bPLS analyses with attention and age were different from those that were correlated with sex and motion. Permutation testing (i.e., resampling without replacement) was completed for each respective analysis (1,000 permutations) to test the significance of the cosine similarity values.

#### Significance, reliability, and reproducibility tests

A total of 1,000 permutations (i.e., resampling “without” replacement) were conducted to test the significance of the PLS results (Fig. 2*C*, left). For each permutation, the singular value was recalculated. The *p* value was then computed as the proportion singular values from the sampling distribution greater than the original singular value. As a reliability test of the weights within each LV, 500 iterations of bootstrap resampling (i.e., resampling “with” replacement) were conducted (Fig. 2*C*, middle). A number of permutations and bootstrapping were determined according to the standards in the literature (Efron and Tibshirani, 1994; Marozzi, 2007). Importantly, bootstrapping suggests that the features of the connectomes assessed are robust. Bootstrap ratios (BSRs) are used to assess the contribution of particular regions or edges to the overall effect. Specifically, BSRs are the ratio of an individual ROI's weight (salience) and standard error of the bootstrap estimates. They are interpreted as a reliability score, similar to *z*-scores where a value of 2.0 corresponds approximately to a 95% confidence interval. BSRs >2.0 therefore indicate the brain regions that reliably contribute to the LV.

Recently, it was demonstrated that performing null hypothesis testing for statistical significance does not assure reproducibility of the results (McIntosh, 2021; Nakua et al., 2024). Therefore, two additional assessments of the reproducibility of the singular values and singular vectors across various sample sizes were conducted (“test-train” and “split-half”, respectively; Fig. 2*C*, right). A “test-train” assessment was performed such that the analysis was performed on random halves of the original sample (split-half resampling) with subjects’ time points held together. The assessment was completed to test if the same LV pattern can produce a similarly strong covariance between *X* and *Y* matrices (i.e., indicated by the singular value). Thus, 500 resamples were performed to produce a distribution of test “singular values”. The distribution's mean and standard deviation were used to compute a *z*-score, whereby a larger value indicates greater reproducibility of the singular values. Moreover, the “split-half” LV reproducibility test was performed in the same way as above (500 random split-half resamples), but the cosine “similarity of singular vectors” was computed. For both assessments, the calculated *z*-scores (from the singular value and left singular vector distributions, respectively) are also compared with the corresponding *z*-scores computed from null distributions generated by permutation resampling (i.e., difference greater than ±2).

#### Code accessibility

The code (run on macOS Big Sur Version 11.5.2) for the computation of the assessed metrics, PLS analyses, and visualization of the key figures are freely available online https://github.com/McIntosh-Lab/Rokos2025_SCFC_NetworkAnalyses and in Extended Data 1.

10.1523/ENEURO.0430-24.2025.d1 Extended Data Supplementary code package with the scripts for computing the metrics, conducting the PLS analyses, and visualisation of the key figures. Download Extended Data, ZIP file.

## Results

### Attention measures

The Wilcoxon's signed rank tests indicated significant performance improvements for each attention task between time points (Table 1). The mixed effects further indicated that age was significantly positively associated with sustained attention (*β* = 2.30; SE = 0.33; *t* = 6.99; *p* < 0.0001), selective attention, (*β* = 1.78; SE = 0.29; *t* = 6.14; *p* < 0.0001), and executive attention (*β* = 4.03; SE = 1.23; *t* = 3.28; *p* = 0.0016). The mixed-model results are also reported in Extended Data Table 1-1.

10.1523/ENEURO.0430-24.2025.t1-1 Table 1-1 Linear mixed-effects models between age and attention. **p < 0.01;***p < 0.001. Download Table 1-1, DOC file.

**Table 1.** Wilcoxon signed rank tests for attention measures between initial and follow-up visits

Measure	Time Point 1 mean (SD)	Time Point 2 mean (SD)	*Z*	*p* value
Sustained attention	23.94 (3.79)	26.78 (2.82)	4.61	*p* < 0.0001
Selective attention	14.15 (3.16)	15.9 (2.45)	3.33	*p* < 0.001
Executive attention	−33.79 (13.22)	−27.62 (6.58)	2.64	*p* = 0.008

The relationship between age and the three attention measures were assessed with Wilcoxon signed rank tests, as well as with linear mixed-effect models (Extended Data Table 1-1).

### Longitudinal change assessments

#### Mean-centered task PLS

In order to explore within-subject changes over the follow-up period, mean-centered task PLS analyses were conducted for each brain measure. The modularity statistics did not change significantly between the baseline and follow-up visits for the SC (*p* = 0.09) or FC matrices (*p* = 0.05). There were no significant longitudinal changes in any of the region-wise SC local clustering (*p* = 0.23) or SC weighted degree (*p* = 0.12) metrics (i.e., no significant LVs identified with permutation testing).

**Figure 3.**
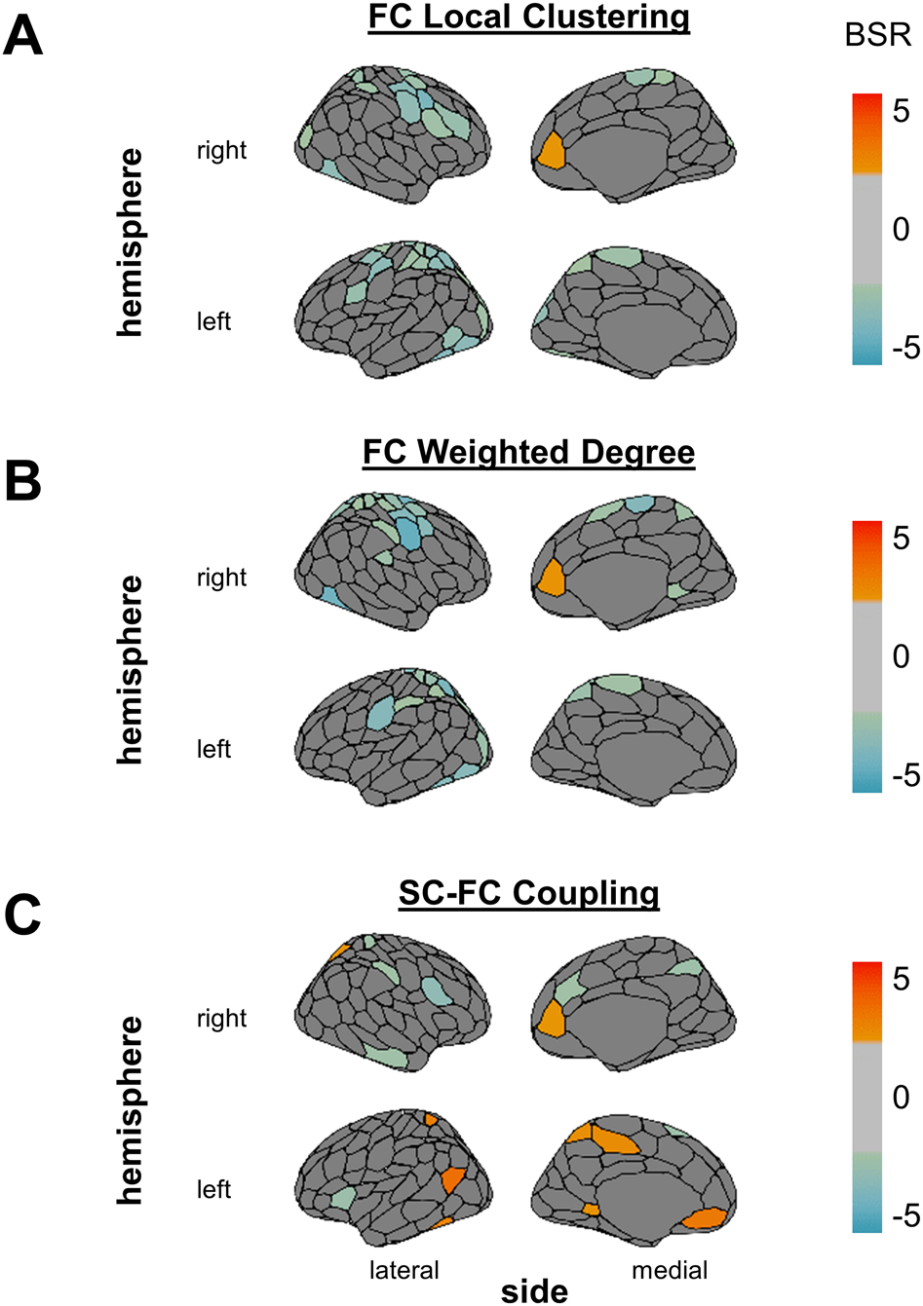
Mean-centered task PLS results. Over the 1 year follow-up period, BSRs indicate regionally specific decreases (negative BSRs, green/blue) and increases (positive BSRs, yellow/red) in FC local clustering (***A***), FC weighted degree** (***B***), and the SC–FC coupling metric (***C***). BSRs thresholded at +2.0 and −2.0 are plotted onto a brain. Analyses that passed the reproducibility tests are marked with **. The cosine similarities between the task PLS analyses and bPLS analyses with potential confounds are outlined in Extended Data Figure 3-1. BSR, bootstrap ratio; SC, structural connectivity; FC, functional connectivity.

The region-wise FC graph metrics exhibited significant alterations over the year, including reduced FC local clustering (*p* = 0.048; *Z*_test-train_ = 1.89; *Z*_null_test-train_ = 0.096; *Z*_split-half_ = 2.71; *Z*_null_split-half_ = 1.45) and weighted degree (*p* = 0.026; *Z*_test-train_ = 2.50; *Z*_null_test-train_ = 0.025; *Z*_split-half_ = 2.94; *Z*_null_split-half_ = 1.39). Several regions showed a decrease in FC local clustering and weighted degree, while the right medial PFC exhibited increases in both metrics (Fig. 3*A*,*B*). Based on the reproducibility tests, only the weighted degree result was reproducible based on the test-train assessment (i.e., *Z* value exceeding the *Z*_null_ value by more than two).

10.1523/ENEURO.0430-24.2025.f3-1 Figure 3-1 Cosine similarities of mean-centred task PLS analyses and behavioural PLS analyses with potential confounds. The cosine similarity of the brain scores and the *p*-values (based on permutation testing) between each mean-centred task PLS analysis and the behavioural PLS analysis of the respective brain metric with sex and motion metrics. Abbreviations: SC = structural connectivity; FC = functional connectivity; LV = latent variable. Download Figure 3-1, DOC file.

The analysis with the SC–FC coupling metric identified one significant LV (*p* = 0.014; *Z*_test-train_ = −0.19; *Z*_null_test-train_ = −0.17; *Z*_split-half_ = 1.32; *Z*_null_split-half_ = 1.30), indicating a regional pattern of increasing [e.g., left and right medial PFC, right superior parietal lobule (SPL), left postcentral gyrus] and decreasing (e.g., right lateral PFC, left insula) coupling between SC and FC (Fig. 3*C*). The reproducibility tests indicated that this relationship was not reproducible.

The cosine similarities between the brain saliences for each task PLS analysis with the respective bPLS analyses with sex and motion, as well as the associated *p* values are reported in Extended Data Figure 3-1. The cosine similarities and permutation testing indicated that there was little overlap between the regions identified by the mean-centered PLS analyses and those associated with sex or motion.

#### Age and longitudinal changes

**Figure 4.**
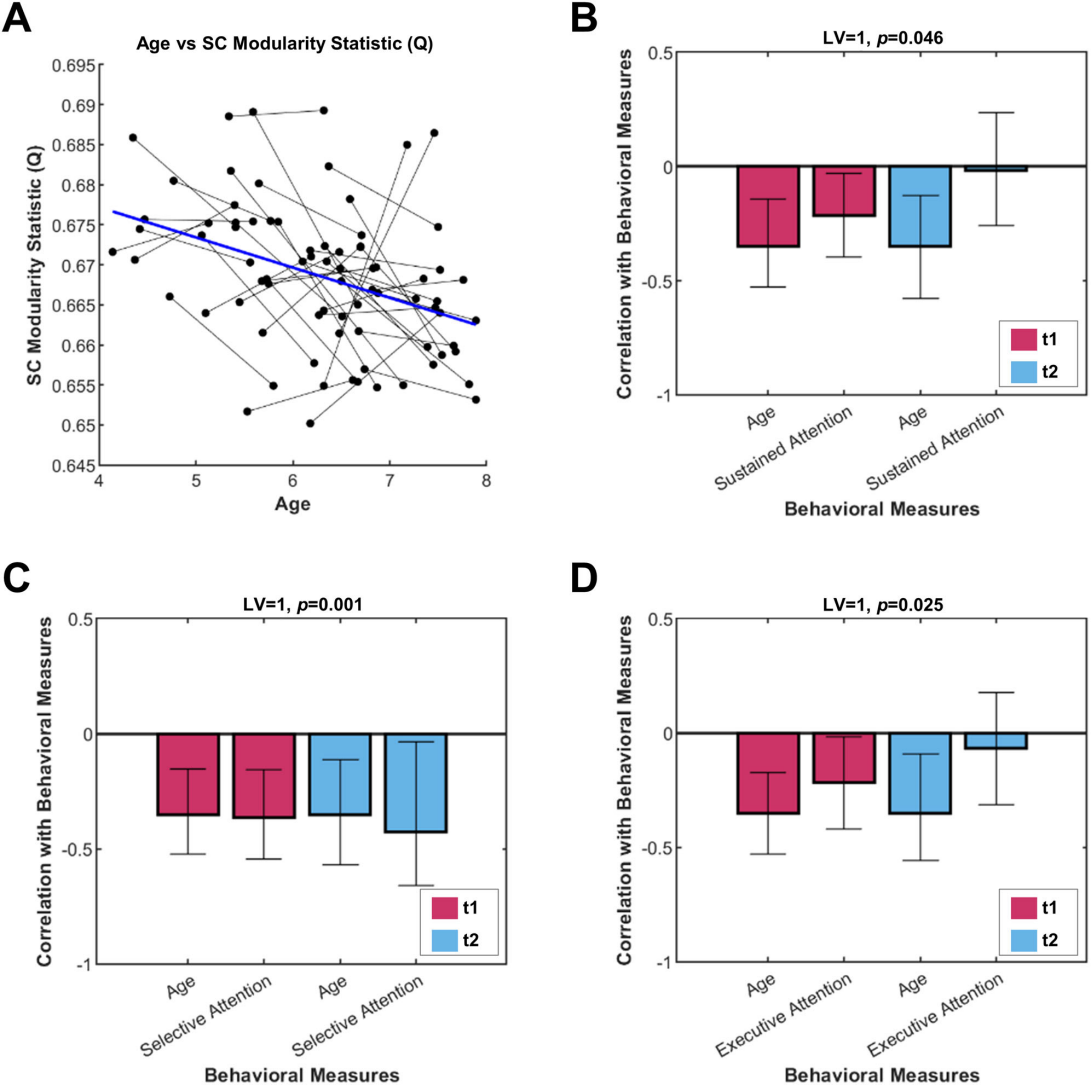
SC modularity results. SC modularity was negatively associated with age (***A***), as well as sustained attention** (***B***), selective attention** (***C***), and executive attention** (***D***), across both the initial and follow-up visits. Analyses that passed the reproducibility tests are marked with **. LV, latent variable; t1, Time Point 1; t2, Time Point 2; SC, structural connectivity.

For the community statistics (*Q*), linear mixed-effect models were also conducted to identify whether changes in modularity were associated with age (with participants as the random intercept). The subject's handedness, sex, visit number, and the dMRI head motion metrics were included to ensure the effects of motion were controlled for. The model indicated that age was negatively associated with SC modularity (*β* = −0.41; SE = 0.14; *t* = −2.92; *p* = 0.006; Fig. 4*A*). The estimated variance by the participant random intercepts was −0.15 (SD = 0.62). FC modularity was not significantly associated with age (*p* = 0.63). The linear models with the differences in *Q* between the two visits as the dependent variable indicated that there were no significant associations with any of the measures (*p*'s > 0.07).

For each significant mean-centered PLS analysis, the correlation between the differences in participants’ brain scores (between time points) with their baseline age were calculated. The correlations were conducted to assess whether the significant longitudinal changes in region-wise metrics (i.e., the region-wise FC metrics, SC–FC coupling) were associated with baseline age. There were no significant correlations between the differences in brain scores and age (|*r|*'s < 0.18; *p*'s > 0.28).

### Cross-sectional assessment—bPLS

A summary of the key results from the mean-centered and bPLS analyses is provided in Table 2. Notably, PLS analyses incorporating all three attention measures and age were conducted. The significant LVs identified by these combined PLS analyses are visualized in Extended Data Table 2-1. The cosine similarity and associated *p* values between the combined and separate attention bPLS analyses revealed significant overlap in the identified relationships (*p*'s < 0.01); thus, we discuss the separate analyses as they serve as tests of simple effects (Extended Data Tables 2-2, 2-3).

10.1523/ENEURO.0430-24.2025.t2-1 Table 2-1 Behavioural PLS analyses with age and three attention measures. The behavioural PLS analyses that included age, as well as sustained, selective and executive attention identified significant latent variables for all structural connectivity (SC) metrics. The identified correlations between SC modularity (***A***), SC weighted degree (***B & C***), and SC local clustering (***D***) with the behavioural measures are plotted. Bootstrap ratios thresholded at +2.0 and -2.0 are plotted onto a brain. Abbreviations: BSR = bootstrap ratio; LV = latent variable; t1 = time point one; t2 = time point two; structural connectivity = SC. Download Table 2-1, TIF file.

10.1523/ENEURO.0430-24.2025.t2-2 Table 2-2 Cosine similarities of structural connectivity local clustering combined and separate behavioural PLS analyses**.** The cosine similarity of the brain scores and the *p*-values (based on permutation testing) between each behavioural PLS (bPLS) analyses of the structural connectivity (SC) local clustering metric with the respective bPLS analyses with all three attention measures and age. Abbreviations: SC = structural connectivity; LV = latent variable. Download Table 2-2, DOC file.

10.1523/ENEURO.0430-24.2025.t2-3 Table 2-3 Cosine similarities of structural connectivity weighted degree combined and separate behavioural PLS analyses**.** The cosine similarity of the brain scores and the *p*-values (based on permutation testing) between each behavioural PLS (bPLS) analyses of the structural connectivity (SC) weighted degree metric with the respective bPLS analyses with all three attention measures and age. Abbreviations: SC = structural connectivity; LV = latent variable. Download Table 2-3, DOC file.

**Table 2.** Summary of the key PLS analyses results

	Mean-centered PLS	Age and sustained attention bPLS	Age and selective attention bPLS	Age and executive attention bPLS	Combined bPLS of SC, FC, and SC–FC coupling with age
Metrics with significant LVs	FC weighted degree	SC modularity	SC modularity	SC modularity	SC weighted degree^b^, FC weighted degree, and SC–FC coupling
	FC local clustering	SC weighted degree^a^	SC weighted degree	SC weighted degree	
	SC–FC coupling	SC local clustering	SC local clustering		

The brain metrics with significant (*p* < 0.05) LVs from the mean-centered PLS analyses and bPLS analyses. Analyses with two significant LVs are denoted with a. The combined analysis with SC weighted degree, FC weighted degree, and SC–FC coupling with age as the dependent variable (combined bPLS of SC, FC, and SC–FC coupling with age) identified SC as the dominant predictor of age, as marked by b. bPLS analyses with age and all three attention measures included were also conducted (Extended Data Table 2-1) and compared with the separate analyses (Extended Data Tables 2-2, 2-3). SC, structural connectivity; FC, functional connectivity.

#### SC modularity

The bPLS analysis between the SC modularity statistic (*Q*) with age and sustained attention revealed a significant LV (*p* = 0.046; *Z*_test-train_ = 2.35; *Z*_null_test-train_ = −0.08; *Z*_split-half_ = 4.39; *Z*_null_split-half_ = 2.21), whereby lower modularity was associated with older age and better sustained attention at time point one. The SC modularity statistic was also negatively associated with age and selective attention at both time points (*p* = 0.001; *Z*_test-train_ = 3.17; *Z*_null_test-train_ = 0.08; *Z*_split-half_ = 8.59; *Z*_null_split-half_ = 2.61) and executive attention at time point one (*p* = 0.025; *Z*_test-train_ = 2.72; *Z*_null_test-train_ = −0.041; *Z*_split-half_ = 4.31; *Z*_null_split-half_ = 2.14). The test-train and split-half resampling tests indicated that these results were reproducible, with *Z* values exceeding the *Z*_null_ values by more than two. The LV with selective attention was significant after Bonferroni’s correction (*α* = 0.05/3). The modularity PLS analyses are visualized in Figure 4*B*–*D*.

#### SC local clustering

**Figure 5.**
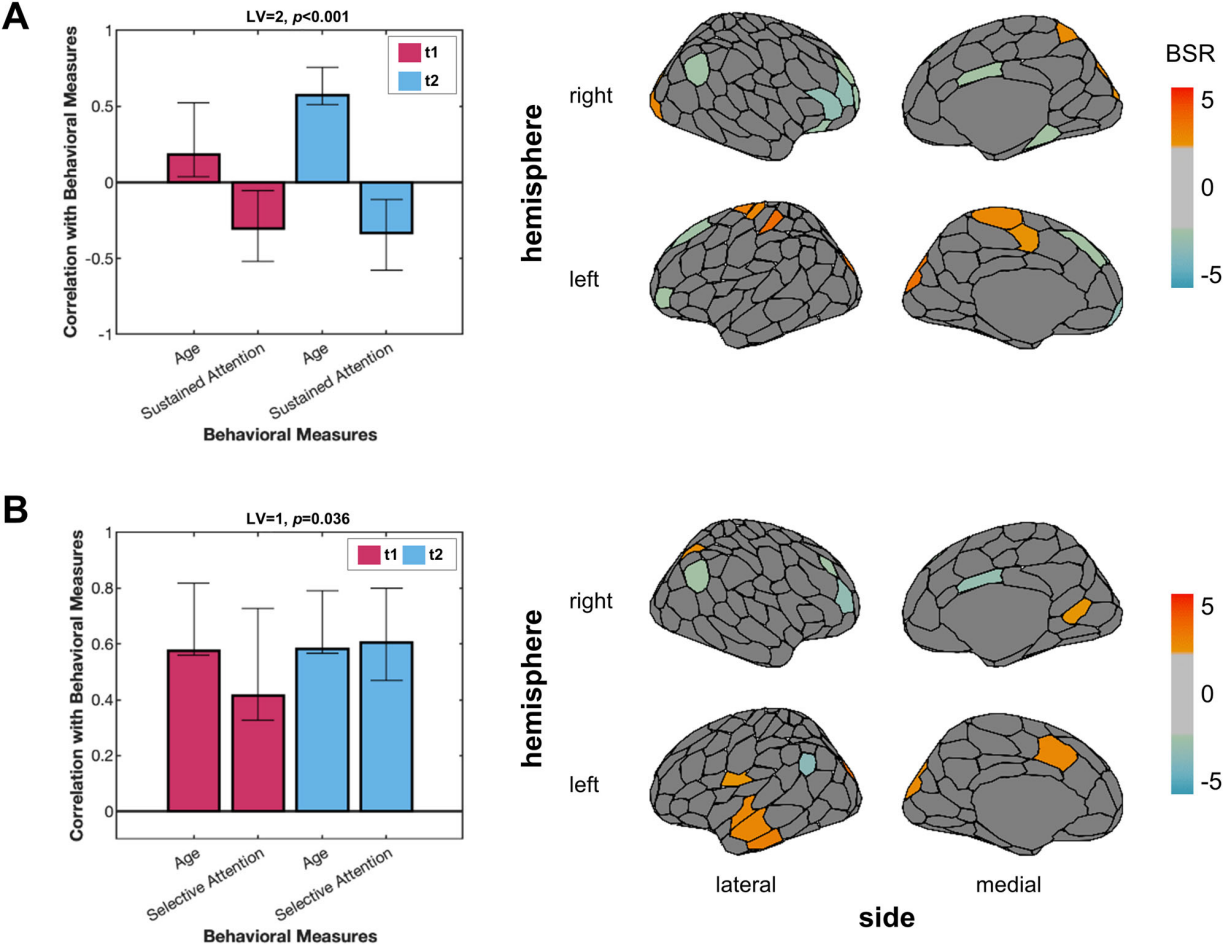
SC local clustering bPLS results. ***A***, The significant LV for the bPLS between SC local clustering with age and sustained attention revealed an age-dependent association. The first LV was not statistically significant. ***B***, The significant LV for the analysis between SC local clustering with age and selective attention revealed that SC local clustering was positively associated with age and selective attention at both time points. BSRs thresholded at +2.0 and −2.0 are plotted onto a brain. The cosine similarities between the SC local clustering bPLS analyses and the bPLS analyses with potential confounds are outlined in Extended Data Figure 5-1. BSR, bootstrap ratio; LV, latent variable; t1, Time Point 1; t2, Time Point 2; SC, structural connectivity.

bPLS analyses for SC local clustering with age and sustained attention identified one significant LV (*p* < 0.001; *Z*_test-train_ = 0.21; *Z*_null_test-train_ = −0.02; *Z*_split-half_ = 1.55; *Z*_null_split-half_ = 1.39). This LV remained significant after Bonferroni’s correction (*α* = 0.05/3). For older participants, worse sustained attention was associated with lower structural local clustering for regions in the default mode network (DMN; e.g., left and right dorsal PFC, right ventral PFC; Fig. 5*A*, negative BSRs), as well as greater local clustering for the right SPL, left somatomotor cortex, and extrastriate regions.

10.1523/ENEURO.0430-24.2025.f5-1 Figure 5-1 Cosine similarities of structural connectivity local clustering behavioural PLS analyses. The cosine similarity of the brain scores and the *p*-values (based on permutation testing) between each behavioural PLS (bPLS) analyses of the structural connectivity (SC) local clustering metric with a) the SC local clustering mean-centred task PLS analysis and b) the bPLS analysis of SC local clustering with sex and motion metrics. Abbreviations: SC = structural connectivity; LV = latent variable. Download Figure 5-1, DOC file.

Furthermore, one significant LV was identified for SC local clustering, age, and selective attention (*p* = 0.036; *Z*_test-train_ = 0.17; *Z*_null_test-train_ = −0.07; *Z*_split-half_ = 1.44; *Z*_null_split-half_ = 1.31; Fig. 5*B*). Regions with greater local clustering, such as the left and right inferior parietal lobule (IPL) and dorsal PFC, were negatively correlated with selective attention and age, at both time points (negative BSRs). Local SC clustering for regions such as the left and right extrastriate cortex, right SPL, and left temporal cortex were positively correlated with age and attention (positive BSRs).

The analysis with SC local clustering, age, and executive attention did not identify any significant LVs (*p* = 0.11). The cosine similarities between the brain saliences from each SC local clustering bPLS (with age and attention) and the respective task PLS analysis and bPLS analysis with potential confounds were computed. The permutation testing for each respective analysis indicated that there was little overlap between the regions that were correlated with age and behavior and those associated with sex or motion (Extended Data Fig. 5-1).

#### SC weighted degree

**Figure 6.**
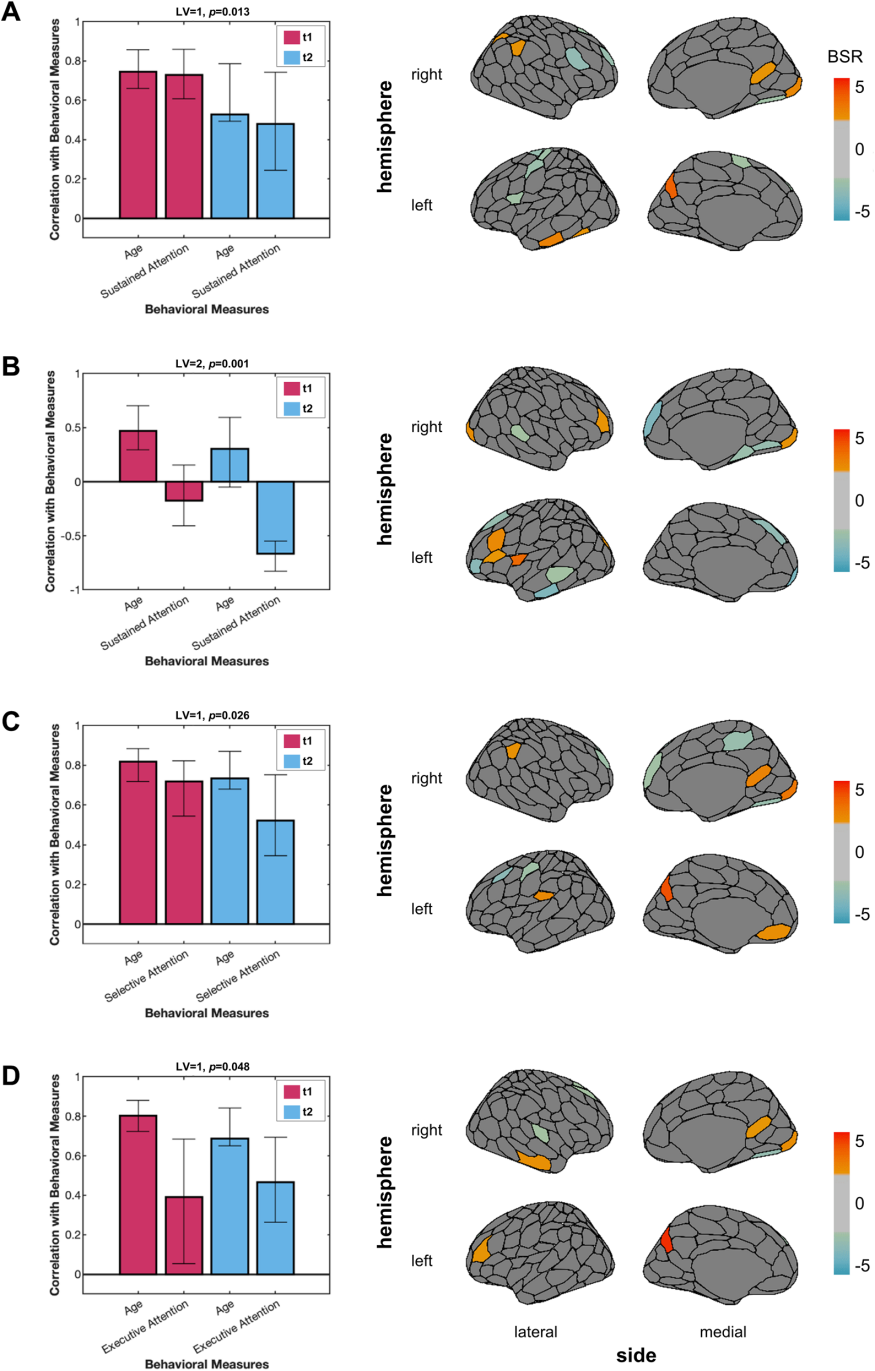
SC weighted degree bPLS results. ***A***, The positive association between SC weighted degree with age and sustained attention, for both time points was evidenced by the first LV. ***B***, A different pattern of brain regions where the relationship between SC weighted degree and sustained attention depended on age was revealed by the second LV. The first LVs for the bPLS analyses between the SC weighted degree with age and selective attention (***C***), and executive attention (***D***), also showed a positive association between SC, age, and attention. BSRs thresholded at +2.0 and −2.0 are plotted onto a brain. The cosine similarities between the SC weighted degree bPLS analyses and the bPLS analyses with potential confounds are outlined in Extended Data Figure 6-1. BSR, bootstrap ratio; LV, latent variable; t1, Time Point 1; t2, Time Point 2; SC, structural connectivity.

The SC weighted degree PLS with age and sustained attention revealed two significant LVs. The first LV (*p* = 0.013; *Z*_test-train_ = 0.013; *Z*_null_test-train_ = 0.0098; *Z*_split-half_ = 1.32; *Z*_null_split-half_ = 1.40) identified regions where weighted degree was positively correlated (e.g., left precuneus, right intraparietal sulcus, right superior and IPL; indicated by positive BSRs), as well as regions where weighted degree was negatively correlated (e.g., left somatomotor cortex, right lateral and dorsolateral PFC; indicated by negative BSRs) with age and sustained attention (Fig. 6*A*). The second LV (*p* = 0.001; *Z*_test-train_ = 0.25; *Z*_null_test-train_ = −0.06; *Z*_split-half_ = 1.24; *Z*_null_split-half_ = 1.32) revealed a unique set of regions where poorer sustained attention was correlated with SC weighted degree for older children. For instance, worse sustained attention in older children was associated with greater weighted degree (positive BSRs) for regions associated with visual processing (e.g., left and right extrastriate) and lower weighted degree (negative BSRs) for regions in the DMN (e.g., left and right medial PFC, left dorsal PFC; Fig. 5*B*). The sustained attention LVs remained significant after Bonferroni’s correction (*α* = 0.05/3).

10.1523/ENEURO.0430-24.2025.f6-1 Figure 6-1 Cosine similarities of structural connectivity weighted degree behavioural PLS analyses. The cosine similarity of the brain scores and the *p*-values (based on permutation testing) between each behavioural PLS (bPLS) analyses of the structural connectivity (SC) weighted degree metric with a) the SC weighted degree mean-centred task PLS analysis and b) the bPLS analysis of SC weighted degree with sex and motion metrics. Abbreviations: SC = structural connectivity; LV = latent variable. Download Figure 6-1, DOC file.

The significant LV which indicated a significant association between SC weighted degree with age and selective attention also revealed a distinct pattern of brain regions at both time points (*p* = 0.026; *Z*_test-train_ = −0.03; *Z*_null_test-train_ = −0.02; *Z*_split-half_ = 1.32; *Z*_null_split-half_ = 1.28; Fig. 6*C*). Specifically, greater weighted degree was associated with better selective attention in older children in regions such as the left precuneus, left secondary somatosensory cortex, and right superior and IPL. Weighted degrees for a number of prefrontal regions (e.g., left and right lateral PFC, right medial PFC, and dorsal PFC) and the medial parietal cortex were negatively correlated with age and selective attention.

An LV indicated a significant association between SC weighted degree with age and executive attention (Fig. 6*D*) independent of the time point (*p* = 0.048; *Z*_test-train_ = −0.50; *Z*_null_test-train_ = −0.004; *Z*_split-half_ = 1.24; *Z*_null_split-half_ = 1.30). In this LV, the left precuneus, left lateral ventral PFC, right striate, right temporal cortex, right retrosplenial cortex, and right SPL were positively correlated with executive function and age, while the right dorsal and dorsolateral PFC, extrastriatal cortex, auditory cortex, and left dorsal PFC were negatively correlated.

The cosine similarities between the brain saliences for the SC weighted degree bPLS analyses (with age and attention), with the respective task PLS analysis and bPLS analysis with sex and motion, indicated that there was also little overlap between the identified regions (Extended Data Fig. 6-1).

#### FC graph metrics

A single significant LV for the FC graph metric weighted degree was identified with age and selective attention (*p* = 0.038); however, the relationship was unstable and weak (i.e., confidence intervals crossed zero, correlations < 0.22). No other significant LVs were observed between the FC metrics, age, and any of the attention measures (*p*'s > 0.08). The cosine similarities between the brain saliences for each FC graph theory bPLS analyses and the respective task PLS analyses indicated that there was little overlap in the regions that differed between the two time points and the regions correlated with age and attention (*p*'s > 0.12). Similarly, the cosine similarities between the brain saliences for the FC bPLS analyses (with age and attention) with the respective bPLS analyses with sex and motion also indicated that there was little overlap between the regions that were correlated with age and behavior and those associated with sex or motion (*p*'s > 0.15).

### SC–FC coupling with age and attention measures

**Figure 7.**
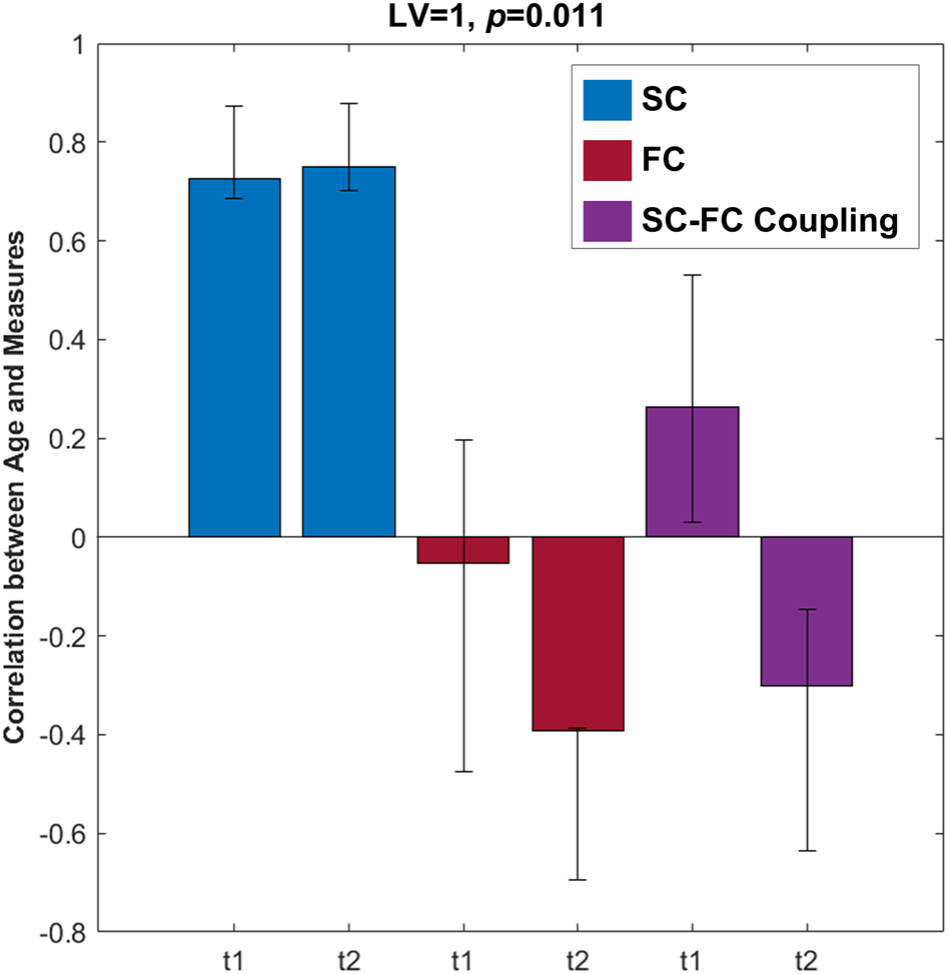
Combined bPLS result with age. The correlation between SC weighted degree, FC weighted degree, and SC–FC coupling with age. The LV with confidence intervals on each of these correlations shows that SC reliably and strongly predicts age, independent of time point. The cosine similarities between the SC–FC coupling bPLS analyses and the bPLS analyses with potential confounds are outlined in Extended Data Figure 7-1. The variance explained by the principal components analyses of the SC and FC matrices are documented in Extended Data Figure 7-2. BSR, bootstrap ratio; LV, latent variable; t1, Time Point 1; t2, Time Point 2; SC, structural connectivity; FC, functional connectivity.

Next, we wanted to explore whether the coupling between SC and FC would collectively identify important brain regions across development. A bPLS analysis was run with SC and FC weighted degree and SC–FC coupling as independent variables and age as the dependent variable. The one significant LV (*p* = 0.011, *Z*_test-train_ = 3.79; *Z*_null_test-train_ = −0.05; *Z*_split-half_ = 9.56; *Z*_null_split-half_ = 1.54) indicated that SC weighted degree was the dominant predictor of age (Fig. 7). The LV identified a negative association between age and SC weighted degree for regions such as the right and left dorsal, medial, and lateral PFC (negative BSRs). Additional bPLS analyses of only SC–FC coupling with age and each attention measure revealed no significant LVs (*p*'s > 0.06).

10.1523/ENEURO.0430-24.2025.f7-1 Figure 7-1 Cosine similarities of SC-FC coupling behavioural PLS analyses. The cosine similarity of the brain scores and the *p*-values (based on permutation testing) between each behavioural PLS (bPLS) analyses with a) the SC-FC coupling mean-centred task PLS analysis and b) the bPLS analysis of SC-FC coupling with sex and motion metrics. Abbreviations: SC = structural connectivity; FC = functional connectivity. Download Figure 7-1, DOC file.

10.1523/ENEURO.0430-24.2025.f7-2 Figure 7-2 Variance explained by the principal components analyses of structural and functional connectivity. The percentage of variance explained by the first ten principal components of the principal components analyses with subjects’ structural and functional connectivity matrices. Download Figure 7-2, DOC file.

The cosine similarities between the brain saliences for the SC–FC coupling PLS analyses with the respective task PLS analyses and bPLS analyses with sex and motion are reported in Extended Data Figure 7-1. The regions identified by the significant bPLS analysis with SC weight degree, FC weighted degree, SC–FC coupling, and age did not overlap highly with those associated with sex or motion (*p* = 0.35).

## Discussion

This study utilized graph analyses to investigate the relationship between both SC and FC with age and attentional abilities during early childhood. Mean-centered task PLS analyses were used to assess longitudinal brain changes in typically developing children between the ages of 4 and 8, a relatively underexplored age range. bPLS analyses were used to explore age associations as well as whether brain network features were related to sustained, selective, and executive attention at each visit.

We identified an association between age and longitudinal developmental decreases in SC modularity (i.e., linear mixed-effect model), as well as associations of lower SC modularity with better attention performance (i.e., bPLS). Network metrics (i.e., local clustering, weighted degree) of the structural networks did not change significantly over the follow-up period but were associated with age and attention at both time points. Two distinct brain–behavior patterns (i.e., LVs) were identified with the bPLS analyses. The first pattern found, across all three attention measures, regions where greater SC weighted degree was associated with older age and better attention. Similarly, greater SC local clustering was associated with better selective attention for older children. The second pattern identified age-dependent relationships for SC local clustering and for SC weighted degree with sustained attention, where the regional SC metrics were associated with worse attention for older children but better attention for younger children.

On the other hand, regional FC graph analyses identified key brain regions that changed between baseline and the 1 year follow-up period but were not associated with age or attention. An additional analysis with SC and FC, as well as SC–FC coupling, further revealed SC as a unique predictor of age during this developmental period. The evident relation between brain network features with attention highlights future opportunities for behavioral interventions or therapies between infancy and middle-to-late childhood (as discussed by Slattery et al., 2022).

### SC

#### SC longitudinal changes

The longitudinal analyses offer a view of brain “development” specifically, beyond the associations with age that can be identified in cross-sectional studies but are at risk of bias (Di Biase et al., 2023). To improve our understanding of brain network development across early childhood, we explored network changes between baseline and the 1 year follow-up period. In line with previous research, we also documented an age-related decrease in SC modularity, signifying a decline in global segregation or greater intermodule integration of structural networks (Hagmann et al., 2010; Dennis et al., 2013; Tymofiyeva et al., 2013; Cao et al., 2017). However, there was no significant association between the changes in modularity with the changes in attention metrics. There were also no significant longitudinal changes in the SC local clustering or weighted degree metrics, suggesting a more stable regional SC topology over the 1 year period investigated in this early childhood timeframe. The longitudinal results are further complemented by the bPLS results which characterized stable structural brain network–behavior associations in early childhood.

#### SC cross-sectional age and attention associations (bPLS)

Although a number of developmental studies have explored age-related differences in structural connectomes, few have assessed their relationship with behavior during early childhood. In order to improve our understanding of how brain network topology is related to behavior in this age range, we investigated the relationship of modularity with attention measures. We demonstrated that lower modularity was associated with better attention in older children. This complements clinical studies where children with ADHD (i.e., worse attention) have been found to exhibit greater modularity than controls and fewer between subnetwork connections (Beare et al., 2017). Importantly, we also expanded upon the characterized global developmental topology of segregation that supports attention by investigating “nodal” metrics.

The LVs for the regional network analyses identified key regions that were associated with age and attention at baseline and follow-up. The cross-sectional SC–behavior relationship and age associations were generally consistent for both time points and are consistent with the longitudinal analyses that found no significant changes in SC regional metrics over the 1 year. The results may be due, in part, to the short follow-up period and can be interpreted as a relationship that is stable or replicated at both visits. For instance, the SC local clustering analyses with selective attention and age, as well as the weighted degree analyses with all attention measures showed positive relationships at both time points. Nonetheless, the cross-sectional analyses allowed for the investigation of age- and attention-related associations over a longer time window (4 years) as opposed to the 1 year follow-up period.

More specifically, reduced SC local clustering of the IPL and prefrontal regions and greater local clustering of the right SPL was associated with older age and better selective attention. The temporal–parietal junction (including the IPL) has shown activation during stimulus-driven attention tasks, involving maintenance, orientation, and reorientation of attention (Igelström and Graziano, 2017). The observed pattern suggests that for older children, lower structural segregation of these regions supports better attention but that segregation of the SPL may be favorable. The right SPL has previously been identified as a critical mediation region for visuospatial attentional tasks (Wu et al., 2016); therefore, interventions that target the right SPL in early childhood may be particularly beneficial for selective attention performance in younger children.

The weighted degree measure also identified the right SPL as a key region where greater average SC strength was associated with better attention and older age. Although there was an overlap in the identified regions between attention tasks (e.g., left precuneus, right SPL), SC weighted degree in unique medial and frontal regions were associated with age and selective attention (e.g., negatively associated with the right medial PFC and medial parietal cortex). Investigating the three different attention measures separately indicated that despite similarities in the regions associated with sustained, selective, and executive attention, they also had unique patterns. Additionally, only sustained attention had an age-dependent relationship with SC local clustering and weighted degree.

#### SC age-dependent relationships with sustained attention

The bPLS analyses identified unique patterns of brain regions where SC local clustering and weighted degree were associated with “worse” sustained attention for older. For example, worse sustained attention for older participants was linked to greater local clustering for a number of extrastriate cortex regions and left somatomotor cortex regions. The SC weighted degree analysis also highlighted a distinct pattern of SC weighted degree associated with poorer sustained attention for older children, including greater weighted degree in visual processing regions and lower weighted degree for PFC regions (i.e., lateral, medial, and dorsal PFC). These results align with early development of sensory regions (Stiles and Jernigan, 2010; Whitaker et al., 2016; Reynolds et al., 2019) and emphasize the significance of age-dependent, region-specific structural segregation for sustained attention performance. Our findings highlight an importance of visual sensory regions for younger children's attention while also indicating that the brain network's continuing reliance on those regions may be unfavorable for older children.

Furthermore, worse sustained attention for older children was associated with greater local clustering for regions associated with the dorsal attention network (DAN; e.g., right SPL and postcentral gyrus), and lower local clustering for the prefrontal DMN (i.e., based on the network structure of the current study's parcellation; Yeo et al., 2011; Schaefer et al., 2018). These findings reinforce the significance of both DAN and DMN regions for sustained attention performance (Esterman et al., 2013). Our results indicate that the influence of specific regions in structural networks on attention is dependent on age and only during a specific window of development. We found that less segregation of communication (i.e., clustering) involving prefrontal DMN regions and particular sensory and attention network regions for older children may negatively impact attention. This is consistent with previous research that has established associations of the DMN with mind-wandering and internally directed processing (Buckner and DiNicola, 2019). The anticorrelation of the DAN and DMN, thought to represent functional segregation of these networks, has also previously been linked with attention problems during preadolescence, with reduced anticorrelation associating with greater attention difficulties (Owens et al., 2020). Therefore, understanding these intricate network changes may yield valuable insights into neurodevelopmental conditions like ADHD, where the balance between small-world properties of DMN, DAN, and visual network regions also appear to be disrupted (Soman et al., 2023).

While the current study is an important step toward better understanding healthy developmental brain networks, future work is required to elucidate how training programs and interventions relate to maturational brain processes (Tymofiyeva and Gaschler, 2021). Graph analyses have been effective in detecting SC changes in children with traumatic brain injury following an intervention designed to improve attention and executive functioning (Yuan et al., 2017). Recent work has also suggested that mindfulness-based training may result in network-specific SC strength increases in healthy adolescents (Tymofiyeva et al., 2024), WM microstructural changes in preterm young adolescents (Siffredi et al., 2023), as well as reduced DMN activation in adolescents with a history of affective disorders (Zhang et al., 2023). In children with ADHD, a brain–computer interface-based attention training game system has also been shown to effectively alter functional brain network topology, with the changes associating with behavioral improvement (Qian et al., 2018). Together, these findings highlight the potential for targeted interventions to modulate SC and FC properties during critical developmental periods.

In summary, two LVs were identified between SC weighted degree, age, and sustained attention. The LVs characterized an intricate association for older children, where worse sustained attention was linked to greater SC weighted degree in primary visual regions, while better sustained attention was associated with greater SC weighted degree in integration regions like the SPL (Molholm et al., 2006). The findings emphasize the relevance and relationship between behavior and the reorganization of structural networks that supports specific regions and networks (e.g., SPL, DAN, DMN) across development. This research underscores the significance of structural network changes in shaping childhood attentional abilities and their potential relevance for addressing age-specific, attention-related challenges in clinical contexts.

### FC

#### FC longitudinal changes

In addition to SC graph metrics, we explored how children's functional networks changed over the 1 year follow-up period. We found that the FC local clustering and weighted degree decreased over the year in numerous visual processing regions and regions associated with the DAN. Interestingly, only the medial PFC exhibited increased local clustering and weighted degree, suggesting its increasing role as a network hub (Hodel, 2018). Cross-sectionally, age-related FC decreases between the DAN and DMN in typically developing children have been reported (Rohr et al., 2017). An increase in self-stability of the functional connectomes over the 1 year follow-up has also been reported to drive greater connectome individualization (i.e., greater longitudinal self-stability relative to between-subject similarity) with age (Graff et al., 2022b). Therefore, along with changes across the follow-up period, we investigated how age was related to FC network development.

On a regional level, the longitudinal changes in the FC metrics were not associated with children's baseline age, indicating a consistent developmental pattern of change across this narrow age range. In line with this, for the bPLS analyses (discussed further below), the FC metrics had no stable cross-sectional brain–behavior relationships and age associations. The identification of only longitudinal changes for FC regional metrics may be attributable to FC demonstrating larger variability between subjects. To explore this, we examined how the variability between the structural and FC matrices differed across subjects via principal component analyses (PCA). The PCA identified that both SC and FC had relatively large first components, indicating a substantial proportion of variance that was common across subjects. Additionally, FC exhibited a smaller first component, and subsequent components explained a larger portion of variance in the FC compared with SC (Extended Data Fig. 7-2). These differences in variance between SC and FC have previously been observed in an aging population (Zimmermann et al., 2019) and highlights the advantages of longitudinal analyses that may be more sensitive to within-subject changes over time. Overall, we characterized a developmental pattern of functional brain network changes over the 1 year follow-up period with no observed functional network–behavior associations in early childhood.

#### FC cross-sectional age and attention associations (bPLS)

The FC graph metric analyses found no significant or reliable attention or age associations. Further investigation with a wider age range would support the characterization of potentially more prominent age-related differences. Rohr et al. (2018) identified distributed functional network integration across this age range. The current study, which focused on FC graph metrics, did not identify age associations, unlike Rohr et al. (2018). However, their study used analyses of specific networks (i.e., independent component analysis) in a larger sample. Future work should also include analyses that capture more specific within-subject changes in brain–behavior relationships (e.g., mixed-effect models), as well as the nonlinear developmental trajectories that have been characterized in a number of network studies (Lebel et al., 2008, 2012). Additionally, dynamic FC has been proposed to be more sensitive to unique aspects of behavior compared with static measures of FC (Eichenbaum et al., 2021). Therefore, characterizing how both static and dynamic FC changes relate to attentional performance across early childhood would allow for an improved understanding of the neural dynamics in the developing brain (Naik et al., 2017). Nonetheless, the results of this study suggest SC topology may play a more substantial, underlying role in attention performance, considering the outsized effect of SC compared with FC and SC–FC coupling in the combined analysis.

### SC–FC coupling

The SC–FC coupling metric was found to change over a year of development, with a region-specific pattern of increasing and decreasing coupling between SC and FC. While the findings highlight the dynamic nature of brain development in early childhood, the changes in coupling were found to have low reproducibility and were not associated with participants’ age. The differences in between-subject variance between structural and FC have also been suggested to limit the subject specificity of the correspondence between SC and FC (Zimmermann et al., 2019).

Similarly, the cross-sectional bPLS analyses between the SC–FC coupling metric with age and each attention measure were nonsignificant. This lack of association might be attributable to the narrow age range of the current sample. Prior research found that the relationship between SC and FC stabilizes and strengthens with age; however these studies considered older and larger age ranges (Betzel et al., 2014; ages 7–85), as well as fewer subjects (Hagmann et al., 2010; *n* = 14, ages 2–18). Furthermore, the current SC–FC coupling approach is a distinction from literature that has focused solely on connections with direct SC links (Soman et al., 2023). All connections were considered due to previous literature suggesting indirect SC may also support FC (Honey et al., 2009).

This study further investigated SC–FC coupling in tandem with SC and FC (weighted degree) and identified SC as the critical predictor of age. Although the SC–FC coupling metric used has previously been shown to reliably predict age for individuals between the ages 18–82 (Zimmermann et al., 2016), the younger sample assessed here may suggest a more distinct independence between SC and FC in early childhood. Another important consideration is that changes in SC appear to precede FC changes (Wendelken et al., 2017; Zuo et al., 2017). This may suggest the possibility of a lead–lag association where SC at early time points predicts changes in later FC and behavior (Wendelken et al., 2017). Furthermore, although the SC–FC coupling metric used in the current study is widely used and straightforward to compute, its simplicity as a linear correlation may not capture the full complexity of the relationship between brain structure and function. For instance, while changes in SC likely play a role in constraining and shaping functional networks, spontaneous and experience-dependent neuronal activity is also thought to influence the development of brain networks (Fair et al., 2007; Honey et al., 2009; Deco et al., 2013; Wang et al., 2013; Mišić et al., 2016). Therefore, computational modeling is a valuable approach that may allow for a better understanding of how SC and FC are “causally” related to each other in this developmental period. Computational frameworks (e.g., The Virtual Brain; Sanz Leon et al., 2013) that integrates multiple neuroimaging modalities (e.g., both SC and FC) and nonlinear network dynamics (e.g., dynamic FC) for typically developing children would provide more extensive insights into healthy brain network trajectories of maturation. Additionally, higher-order connectomics, which consider interactions among three or more regions simultaneously, have recently been applied in adults to infer complex brain network interactions and may offer greater sensitivity for detecting brain–behavior associations compared with pairwise statistics (Santoro et al., 2024). Thus, more advanced and complex methods that account for interactions among multiple brain regions, scales, and modalities may be better suited for studying the intricate relationship between structure and function during development.

### Important considerations and limitations

The test-train and split-half resampling approaches described in this study emphasize the importance of considering the level of reproducibility, in addition to the reliability (bootstrapping) and significance (permutation testing) of each analysis (Nakua et al., 2024). For example, a number of significant brain–behavior relationships were not reproducible, likely due to the small sample size analyzed. In order to improve the generalizability of the findings, future work should consider data collected from multiple sites, a larger sample, wider age range, and follow-ups beyond 1 year. The smaller sample size analyzed was in part due to rigorous data processing/cleaning and QC to mitigate the potential impact of motion (Power et al., 2012; Grayson and Fair, 2017; Oldham and Fornito, 2019).

The passive-viewing functional scans may be confounded by the level of attentiveness and enjoyment of the show during the scan, which would add more intersubject variability (Rohr et al., 2016); therefore subsequent studies should consider including a measure to assess participants’ level of engagement. As discussed previously (Rohr et al., 2018), the behavioral attention measures may also be influenced by situational context (e.g., children may prioritize their attention differently depending on the context) and may be limited in terms of real-world applicability. Lastly, the current parcellation only includes cortical regions (due to the likelihood of increased false positive connections within subcortical regions); however atypical frontosubcortical connectivity has been displayed in children with ADHD (Kumar et al., 2022; Rosch et al., 2023). In order to achieve a more comprehensive understanding of the interplay between the whole-brain structure–function relationship with attention, future work should explore whether the results are impacted by the inclusion of subcortical regions and the use of different parcellation approaches.

### Conclusion

In children aged 4–8 years, region-wise graph analyses supported the characterization of variable developmental changes and brain–behavior relationships across the brain. Over a 1 year follow-up period, longitudinal changes in functional graph metrics were observed, as well as age-related decreases in SC modularity. This study further emphasizes how structural topology is related to age and attentional performance. The local clustering and weighted degree metrics identified key regions where lower SC segregation was associated with better selective attention skills in older children but also differentiated regions (e.g., right SPL) where greater SC weighted degree and clustering appeared to be beneficial. Evidently, early childhood is an extremely dynamic period where cognitive functioning is intricately and predominantly linked to structural network features. The current findings carry numerous implications for understanding healthy development and identifying potential targets for neurodevelopmental disorders.

## Synthesis

Reviewing Editor: Arvind Kumar, KTH Royal Institute of Technology Decisions are customarily a result of the Reviewing Editor and the peer reviewers coming together and discussing their recommendations until a consensus is reached. When revisions are invited, a fact-based synthesis statement explaining their decision and outlining what is needed to prepare a revision will be listed below. The following reviewer(s) agreed to reveal their identity: Pascal Helson.

Synthesis

With this manuscript authors advance our understanding of how network properties evolve in early childhood and how they may be related to attention behavior. The work is highly relevant and it brings new methods to study brain development. However the two reviewers think that the manuscript needs further clarification on the methods and analysis pipeline. Moreover, they have made several suggestions to improve the work. In the following I have appended the comments from the two reviewers. We look forward to the revision of the manuscript.

Reviewer #1

This is an interesting paper on a topic of relevance, structure-function evolution during neurodevelopment in association with behaviour, well conducted and well formulated.

The analysis conducted appears rigorous and very complete.

The manuscript is very well written, clear and exhaustive.

I have the following remarks and suggestions, mostly regarding the methodological implementation:

- Lines 316-317: The description of the computation for structure-function coupling as it is now, states that a correlation was computed between two "degree vectors", computed for each subject, so this would yield one value per subject, and not per region, as pointed out in the next sentence. I think therefore this could be a correlation between regional connectivity profiles (instead of degree vectors?), computed for each region? Please clarify this in the text.

- The way to compute structure-function coupling is very basic (one-to-one correlation between SC and FC). It was already highlighted in the past that the relationship between brain structure and function appears more complex than a linear one-to-one correlation, and more advanced methods were introduced recently to analyse this relationship in adults. Please discuss this in the manuscript, with reference to the relevant literature. (advantages of simpler formulation? Limitation?)

- PLS: If I understand correctly, different PLS analyses were conducted for each behavioral variable (sustained / selective / executive attention)? Why not include them all in the same PLS analysis?

-Again about PLS: It seems to me that for the current formulation of the PLS, cross-sectional effects of age rather than longitudinal ones are examined. Since the two timepoints are treated as two unpaired groups, with no information about within subject variability? Recently, the longitudinal effect of age on structure-function coupling has been explored in a repeated analysis with PLS in this paper: https://www.sciencedirect.com/science/article/pii/S2451902224001411.

Please explore this method (age coded as mean-difference-interaction) and see if that could be more reflecting your need in highlighting a longitudinal effect. Reformulate clarifying the ceross-sectional nature of the age-effect explored, otherwise.

Reviewer #2

In this paper, the authors study how segregation network measures of functional (FC) and structural connectivity (SC) relate to age and attention in early childhood (ages 4-8). In addition, a one-year longitudinal analysis is performed. The main result is the presence of covariability of the SC segregation network measures and age and attention performances but not in the FC. Interestingly, this covariability sign may differ depending on the brain region pointing towards regions where segregation increase (or decrease) should be targeted in helping children with attention disorders. Longitudinal changes were observed in both FC and SC metrics.

In general, the manuscript is well written, and I really appreciated the clear data preprocessing/cleaning description. However, the analysis deserves additional details to help the reader follow and interpret the results. Hence, mainly regarding the analysis part, I have several questions, comments, and suggestions that I hope will help improve the manuscript. I will insist on what follows on the general comments and more specific ones can be found in the attached marked documents (see separate files).

First, in the introduction, there is a need to argue on why you chose the specific network measures. For example, why only segregation and not integration measures?

Second, a figure detailing the analysis pipeline would greatly help. It should show the main steps: from SC/FC matrices to network measures matrices (X) then combined with the behaviour/task matrix (Y) to get latent variables (L_X/Y) and the correlation vectors between behaviour (Y) and the brain latent variable (L_X), then permutation and bootstrapping to finally get the statistics used in the plots (BSR for example), give an example plot then (brain plus correlation plot). In addition, a table with the main results would help a lot following the discussion.

Third, regarding the longitudinal analysis, it would be more efficient to look at the differences between time points and perform a SVD on Y_diff^T X_diff. Indeed, with the mean-centered task PLS you lose the individual changes and mix them together. Importantly, for the mean-centered task PLS on modularity, you are comparing two points in 1D (on a line) ... A linear mixed model would make more sense in this case where you don't have region-wise values.

Fourth, your discussion on DAN and DMN (interesting in my opinion) needs to be based on further analysis, as such it is not convincing. Also, does there exist a protocol to change the SC and FC properties as you propose to do on ADHD children? Please develop this point which is (for me) one of the most valuable parts of your work.

Finally, a technical point, please check that the algorithm is z-scoring the matrices (column-wise) which is essential for interpreting the saliences as correlations.
