# Supplementary material for: Examining Relationships between Functional and Structural Brain Network Architecture, Age, and Attention Skills in Early Childhood
Source: eNeuro. 2025 Jul 24;12(7):ENEURO.0430-24.2025. doi: 10.1523/ENEURO.0430-24.2025 (PMC12320921; doi:10.1523/ENEURO.0430-24.2025)
Supplement: Figure 2-1 — Parcellation region abbreviations and full names. Download Figure 2-1, DOC file. [file eneuro-12-ENEURO.0430-24.2025-s007.doc]

**Extended Data Figure 2-1. Parcellation region abbreviations and full names**

| Region Abbreviation | Region Full Name | Region Abbreviation | Region Full Name |
| --- | --- | --- | --- |
| AntTemp | anterior temporal | PFCd | dorsal prefrontal cortex |
| Aud | auditory | PFCl | lateral prefrontal cortex |
| Cent | central | PFCld | lateral dorsal prefrontal cortex |
| Cinga | cingulate anterior | PFClv | lateral ventral prefrontal cortex |
| Cingm | mid-cingulate | PFCm | medial prefrontal cortex |
| Cingp | cingulate posterior | PFCmp | medial posterior prefrontal cortex |
| ExStr | extrastriate cortex | PFCv | ventral prefrontal cortex |
| ExStrInf | extra-striate inferior | PHC | parahippocampal cortex |
| ExStrSup | extra-striate superior | PostC | post central |
| FEF | frontal eye fields | PrC | precentral |
| FPole | frontal pole | PrCd | precentral dorsal |
| FrMed | frontal medial | PrCv | precentral ventral |
| FrOper | frontal operculum | RSC | retrosplenial cortex |
| IFG | inferior frontal gyrus | Rsp | retrosplenial |
| Ins | insula | S2 | S2 |
| IPL | inferior parietal lobule | SPL | superior parietal lobule |
| IPS | intraparietal sulcus | ST | superior temporal |
| OFC | orbital frontal cortex | Striate | striate cortex |
| ParMed | parietal medial | StriCal | striate calcarine |
| ParOcc | parietal occipital | Temp | temporal |
| ParOper | parietal operculum | TempOcc | temporal occipital |
| pCun | precuneus | TempPar | temporal parietal |
| pCunPCC | precuneus posterior cingulate cortex | TempPole | temporal pole |
