# Supplementary material for: Examining Relationships between Functional and Structural Brain Network Architecture, Age, and Attention Skills in Early Childhood
Source: eNeuro. 2025 Jul 24;12(7):ENEURO.0430-24.2025. doi: 10.1523/ENEURO.0430-24.2025 (PMC12320921; doi:10.1523/ENEURO.0430-24.2025)
Supplement: Figure 2-2 — Region name and parcellation number for the regions excluded from the analyses. Download Figure 2-2, DOC file. [file eneuro-12-ENEURO.0430-24.2025-s008.doc]

**Extended Data Figure 2-2. Region name and parcellation number for the regions excluded from the analyses**

| Region Name | Region Number |
| --- | --- |
| 17Networks_LH_LimbicA_TempPole_1 | 53 |
| 17Networks_LH_LimbicA_TempPole_2 | 54 |
| 17Networks_LH_LimbicA_TempPole_3 | 55 |
| 17Networks_LH_DefaultB_Temp1 | 83 |
| 17Networks_RH_LimbicA_TempPole_1 | 161 |
| 17Networks_RH_LimbicA_TempPole_2 | 162 |
| 17Networks_RH_LimbicA_TempPole_3 | 163 |
|  |  |
