# Supplementary material for: Examining Relationships between Functional and Structural Brain Network Architecture, Age, and Attention Skills in Early Childhood
Source: eNeuro. 2025 Jul 24;12(7):ENEURO.0430-24.2025. doi: 10.1523/ENEURO.0430-24.2025 (PMC12320921; doi:10.1523/ENEURO.0430-24.2025)
Supplement: Table 1-1 — Linear mixed-effects models between age and attention. **p < 0.01;***p < 0.001. Download Table 1-1, DOC file. [file eneuro-12-ENEURO.0430-24.2025-s003.doc]

**Extended Data Table 1-1. Linear mixed-effects models between age and attention**

|  | Sustained Attention | Selective Attention | Executive Attention |
| --- | --- | --- | --- |
| Random Effects  Variance (SD) | | | |
| Participants (intercept) | 6.38 (2.53) | 1.68 (1.30) | 9.02 (3.00) |
| Residual | 4.03 (2.01) | 4.14 (2.03) | 95.54 (9.77) |
| Fixed Effect  β (SE, t-value) | | | |
| Intercept | 10.22 (2.20, 4.66) | 3.83 (1.85, 2.07) | -56.16 (7.87, -7.14) |
| Age | 2.39 (0.34, 7.04)*** | 1.78 (0.29, 6.14)*** | 4.04 (1.23, 3.38)** |

***p*<0.01;****p*<0.001
