# Supplementary material for: Examining Relationships between Functional and Structural Brain Network Architecture, Age, and Attention Skills in Early Childhood
Source: eNeuro. 2025 Jul 24;12(7):ENEURO.0430-24.2025. doi: 10.1523/ENEURO.0430-24.2025 (PMC12320921; doi:10.1523/ENEURO.0430-24.2025)
Supplement: Figure 3-1 — Cosine similarities of mean-centred task PLS analyses and behavioural PLS analyses with potential confounds. The cosine similarity of the brain scores and the p-values (based on permutation testing) between each mean-centred task PLS analysis and the behavioural PLS analysis of the respective brain metric with sex and motion metrics. Abbreviations: SC = structural connectivity; FC = functional connectivity; LV = latent variable. Download Figure 3-1, DOC file. [file eneuro-12-ENEURO.0430-24.2025-s009.doc]

**Extended Data Figure 3-1. Cosine similarities of mean-centred task PLS analyses and behavioural PLS analyses with potential confounds**

| Measure | Cosine Similarity with Potential Confounds Behavioural PLS | *p*-value |
| --- | --- | --- |
|  |  |  |
| SC Local Clustering | -0.11 | 0.47 |
| SC Weighted Degree | 0.25 | 0.13 |
| FC Local Clustering | 0.54 | 0.50 |
| FC Weighted Degree | 0.49 | 0.49 |
| SC-FC Coupling | 0.02 | 0.81 |

The cosine similarity of the brain scores and the *p*-values (based on permutation testing) between each mean-centred task PLS analysis and the behavioural PLS analysis of the respective brain metric with sex and motion metrics. Abbreviations: SC = structural connectivity; FC = functional connectivity; LV = latent variable.
