# Supplementary figures and images for: Examining Relationships between Functional and Structural Brain Network Architecture, Age, and Attention Skills in Early Childhood
Source: eNeuro. 2025 Jul 24;12(7):ENEURO.0430-24.2025. doi: 10.1523/ENEURO.0430-24.2025 (PMC12320921; doi:10.1523/ENEURO.0430-24.2025)

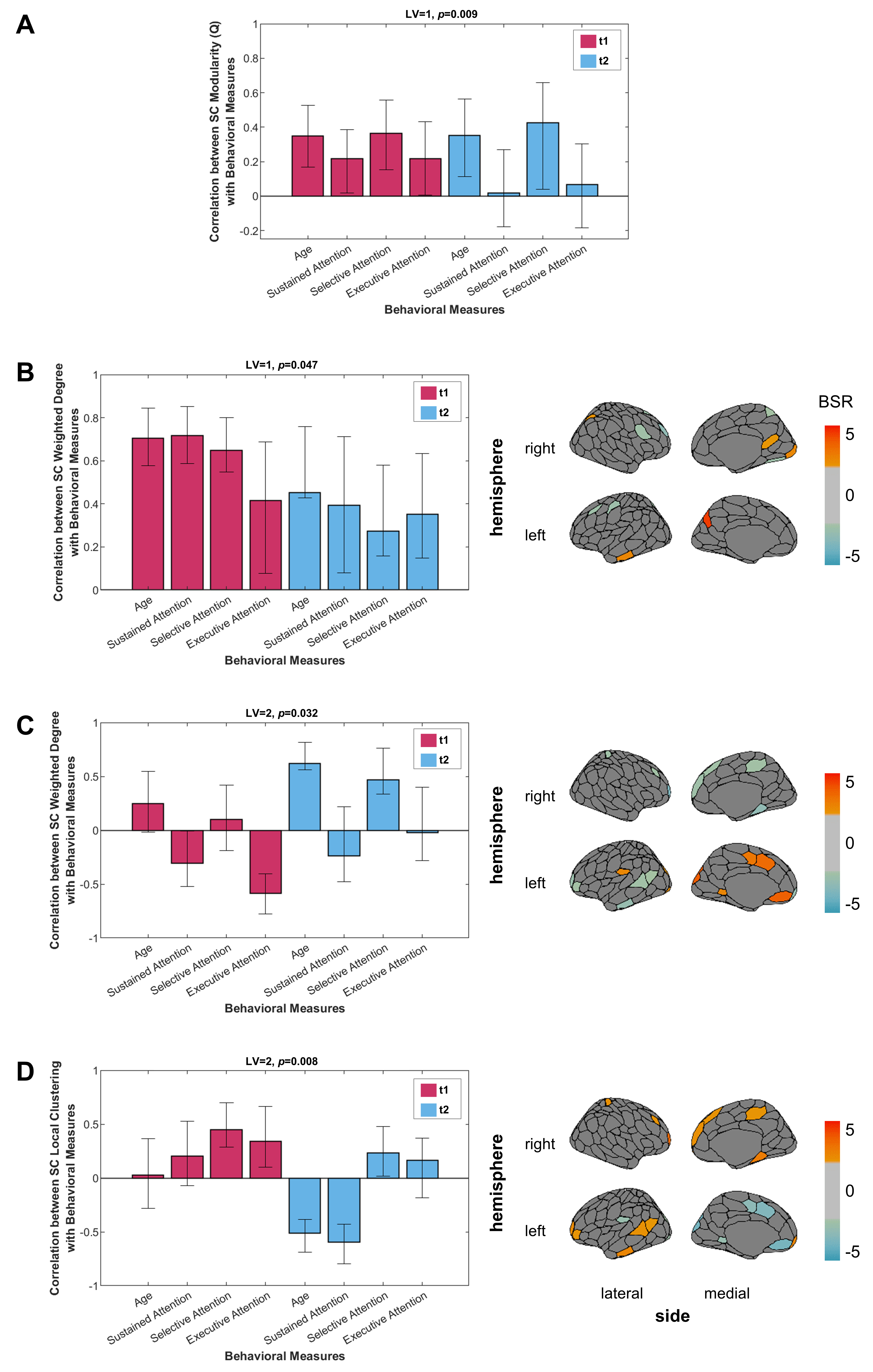

Supplement: Table 2-1 — Behavioural PLS analyses with age and three attention measures. The behavioural PLS analyses that included age, as well as sustained, selective and executive attention identified significant latent variables for all structural connectivity (SC) metrics. The identified correlations between SC modularity (A), SC weighted degree (B & C), and SC local clustering (D) with the behavioural measures are plotted. Bootstrap ratios thresholded at +2.0 and -2.0 are plotted onto a brain. Abbreviations: BSR = bootstrap ratio; LV = latent variable; t1 = time point one; t2 = time point two; structural connectivity = SC. Download Table 2-1, TIF file. [file eneuro-12-ENEURO.0430-24.2025-s004.tif]
