# Supplementary material for: Examining Relationships between Functional and Structural Brain Network Architecture, Age, and Attention Skills in Early Childhood
Source: eNeuro. 2025 Jul 24;12(7):ENEURO.0430-24.2025. doi: 10.1523/ENEURO.0430-24.2025 (PMC12320921; doi:10.1523/ENEURO.0430-24.2025)
Supplement: Table 2-3 — Cosine similarities of structural connectivity weighted degree combined and separate behavioural PLS analyses. The cosine similarity of the brain scores and the p-values (based on permutation testing) between each behavioural PLS (bPLS) analyses of the structural connectivity (SC) weighted degree metric with the respective bPLS analyses with all three attention measures and age. Abbreviations: SC = structural connectivity; LV = latent variable. Download Table 2-3, DOC file. [file eneuro-12-ENEURO.0430-24.2025-s006.doc]

**Extended Data Table 2-3. Cosine similarities of structural connectivity weighted degree combined and separate behavioural PLS analyses**

| Measure | Cosine Similarity with Combined bPLS – SC Weighted Degree LV1 | *p-*value | Cosine Similarity with Combined bPLS – SC Weighted Degree LV2 | *p*-value |
| --- | --- | --- | --- | --- |
| SC Weighted Degree – Sustained Attention | 0.95 | <0.0001 | 0.60 | <0.0001 |
| SC Weighted Degree – Selective Attention | 0.86 | <0.0001 | 0.60 | <0.0001 |
| SC Weighted Degree – Executive Attention | 0.87 | <0.0001 | 0.83 | <0.0001 |

The cosine similarity of the brain scores and the *p*-values (based on permutation testing) between each behavioural PLS (bPLS) analyses of the structural connectivity (SC) weighted degree metric with the respective bPLS analyses with all three attention measures and age. Abbreviations: SC = structural connectivity; LV = latent variable.
