# Supplementary material for: Examining Relationships between Functional and Structural Brain Network Architecture, Age, and Attention Skills in Early Childhood
Source: eNeuro. 2025 Jul 24;12(7):ENEURO.0430-24.2025. doi: 10.1523/ENEURO.0430-24.2025 (PMC12320921; doi:10.1523/ENEURO.0430-24.2025)
Supplement: Figure 5-1 — Cosine similarities of structural connectivity local clustering behavioural PLS analyses. The cosine similarity of the brain scores and the p-values (based on permutation testing) between each behavioural PLS (bPLS) analyses of the structural connectivity (SC) local clustering metric with a) the SC local clustering mean-centred task PLS analysis and b) the bPLS analysis of SC local clustering with sex and motion metrics. Abbreviations: SC = structural connectivity; LV = latent variable. Download Figure 5-1, DOC file. [file eneuro-12-ENEURO.0430-24.2025-s010.doc]

**Extended Data Figure 5-1. Cosine similarities of structural connectivity local clustering behavioural PLS analyses**

| Measure | Cosine Similarity with Task PLS | *p-*value | Cosine Similarity with Potential Confounds Behavioural PLS | *p*-value |
| --- | --- | --- | --- | --- |
| SC Local Clustering - Sustained Attention LV2 | 0.06 | 0.61 | 0.11 | 0.70 |
| SC Local Clustering - Selective Attention | -0.07 | 0.65 | 0.20 | 0.51 |
| SC Local Clustering - Executive Attention | -0.009 | 0.93 | 0.11 | 0.73 |

The cosine similarity of the brain scores and the *p*-values (based on permutation testing) between each behavioural PLS (bPLS) analyses of the structural connectivity (SC) local clustering metric with a) the SC local clustering mean-centred task PLS analysis and b) the bPLS analysis of SC local clustering with sex and motion metrics. Abbreviations: SC = structural connectivity; LV = latent variable.
