# Supplementary material for: Examining Relationships between Functional and Structural Brain Network Architecture, Age, and Attention Skills in Early Childhood
Source: eNeuro. 2025 Jul 24;12(7):ENEURO.0430-24.2025. doi: 10.1523/ENEURO.0430-24.2025 (PMC12320921; doi:10.1523/ENEURO.0430-24.2025)
Supplement: Figure 6-1 — Cosine similarities of structural connectivity weighted degree behavioural PLS analyses. The cosine similarity of the brain scores and the p-values (based on permutation testing) between each behavioural PLS (bPLS) analyses of the structural connectivity (SC) weighted degree metric with a) the SC weighted degree mean-centred task PLS analysis and b) the bPLS analysis of SC weighted degree with sex and motion metrics. Abbreviations: SC = structural connectivity; LV = latent variable. Download Figure 6-1, DOC file. [file eneuro-12-ENEURO.0430-24.2025-s011.doc]

**Extended Data Figure 6-1. Cosine similarities of structural connectivity weighted degree behavioural PLS analyses**

| Measure | Cosine Similarity with Task PLS | *p*-value | Cosine Similarity with Potential Confounds Behavioural PLS | *p*-value |
| --- | --- | --- | --- | --- |
| SC Weighted Degree - Sustained Attention LV1 | -0.03 | 0.84 | -0.15 | 0.53 |
| SC Weighted Degree - Sustained Attention LV2 | 0.09 | 0.49 | 0.32 | 0.074 |
| SC Weighted Degree - Selective Attention | -0.07 | 0.60 | -0.038 | 0.85 |
| SC Weighted Degree - Executive Attention | -0.02 | 0.89 | 0.023 | 0.91 |

The cosine similarity of the brain scores and the *p*-values (based on permutation testing) between each behavioural PLS (bPLS) analyses of the structural connectivity (SC) weighted degree metric with a) the SC weighted degree mean-centred task PLS analysis and b) the bPLS analysis of SC weighted degree with sex and motion metrics. Abbreviations: SC = structural connectivity; LV = latent variable.
