# Supplementary material for: Examining Relationships between Functional and Structural Brain Network Architecture, Age, and Attention Skills in Early Childhood
Source: eNeuro. 2025 Jul 24;12(7):ENEURO.0430-24.2025. doi: 10.1523/ENEURO.0430-24.2025 (PMC12320921; doi:10.1523/ENEURO.0430-24.2025)
Supplement: Figure 7-1 — Cosine similarities of SC-FC coupling behavioural PLS analyses. The cosine similarity of the brain scores and the p-values (based on permutation testing) between each behavioural PLS (bPLS) analyses with a) the SC-FC coupling mean-centred task PLS analysis and b) the bPLS analysis of SC-FC coupling with sex and motion metrics. Abbreviations: SC = structural connectivity; FC = functional connectivity. Download Figure 7-1, DOC file. [file eneuro-12-ENEURO.0430-24.2025-s012.doc]

**Extended Data Figure 7-1. Cosine similarities of SC-FC coupling behavioural PLS analyses**

| Measure | Cosine Similarity with Task PLS | *p*-value | Cosine Similarity with Potential Confounds Behavioural PLS | *p*-value |
| --- | --- | --- | --- | --- |
| SC-FC Coupling - Sustained Attention | 0.08 | 0.51 | 0.063 | 0.74 |
| SC-FC Coupling - Selective Attention | 0.07 | 0.83 | 0.63 | 0.031 |
| SC-FC Coupling - Executive Attention | 0.06 | 0.77 | -0.30 | 0.21 |
| SC-FC Coupling, SC Weighted Degree, FC Weighted Degree – Age | NA | NA | 0.14 | 0.35 |

The cosine similarity of the brain scores and the *p*-values (based on permutation testing) between each behavioural PLS (bPLS) analyses with a) the SC-FC coupling mean-centred task PLS analysis and b) the bPLS analysis of SC-FC coupling with sex and motion metrics. Abbreviations: SC = structural connectivity; FC = functional connectivity.
