# Supplementary material for: Examining Relationships between Functional and Structural Brain Network Architecture, Age, and Attention Skills in Early Childhood
Source: eNeuro. 2025 Jul 24;12(7):ENEURO.0430-24.2025. doi: 10.1523/ENEURO.0430-24.2025 (PMC12320921; doi:10.1523/ENEURO.0430-24.2025)
Supplement: Figure 7-2 — Variance explained by the principal components analyses of structural and functional connectivity. The percentage of variance explained by the first ten principal components of the principal components analyses with subjects’ structural and functional connectivity matrices. Download Figure 7-2, DOC file. [file eneuro-12-ENEURO.0430-24.2025-s013.doc]

**Extended Data Figure 7-2. Variance explained by the principal components analyses of structural and functional connectivity**

| Principal Component | Structural Connectivity Percentage of Variance Explained | Functional Connectivity Percentage of Variance Explained |
| --- | --- | --- |
| 1 | 89.06 | 67.50 |
| 2 | 0.45 | 1.74 |
| 3 | 0.36 | 1.14 |
| 4 | 0.34 | 1.04 |
| 5 | 0.32 | 0.94 |
| 6 | 0.30 | 0.87 |
| 7 | 0.29 | 0.80 |
| 8 | 0.29 | 0.78 |
| 9 | 0.28 | 0.73 |
| 10 | 0.26 | 0.72 |

The percentage of variance explained by the first ten principal components of the principal components analyses with subjects’ structural and functional connectivity matrices.
